# Novel bright-emission small-molecule NIR-II fluorophores for *in vivo* tumor imaging and image-guided surgery†
†Electronic supplementary information (ESI) available. See DOI: 10.1039/c7sc00251c



**DOI:** 10.1039/c7sc00251c

**Published:** 2017-02-21

**Authors:** Yao Sun, Mingmin Ding, Xiaodong Zeng, Yuling Xiao, Huaping Wu, Hui Zhou, Bingbing Ding, Chunrong Qu, Wei Hou, AGA Er-bu, Yejun Zhang, Zhen Cheng, Xuechuan Hong

**Affiliations:** a State Key Laboratory of Virology , Key Laboratory of Combinatorial Biosynthesis and Drug Discovery (MOE) , Hubei Provincial Key Laboratory of Developmentally Originated Disease , Wuhan University School of Pharmaceutical Sciences , Wuhan 430071 , China . Email: xhy78@whu.edu.cn; b Key Laboratory of Pesticides and Chemical Biology , Ministry of Education , College of Chemistry , Central China Normal University , Wuhan 430079 , China; c Molecular Imaging Program at Stanford (MIPS) , Bio-X Program , Department of Radiology , Stanford University , California 94305-5344 , USA; d Medical College , Tibet University , Lasa , 850000 , China; e Suzhou NIR-Optics Technologies Co., Ltd , Suzhou , 215123 , China

## Abstract

Though high brightness and biocompatible small NIR-II dyes are highly desirable in clinical or translational cancer research, their fluorescent cores are relatively limited and their synthetic processes are somewhat complicated. Herein, we have explored the design and synthesis of novel NIR-II fluorescent materials (**H1**) without tedious chromatographic isolation with improved fluorescence performance (QY ≈ 2%) by introducing 2-amino 9,9-dialkyl-substituted fluorene as a donor into the backbone. Several types of water-soluble and biocompatible NIR-II probes: **SXH**, **SDH**, and **H1 NPs** were constructed *via* different chemical strategies based on **H1**, and then their potential to be used in *in vivo* tumor imaging and image-guided surgery in the NIR-II region was explored. High levels of uptake were obtained for both passive and active tumor targeting probes **SXH** and **SDH**. Furthermore, high resolution imaging of blood vessels on tumors and the whole body of living mice using **H1 NPs** for the first time has demonstrated precise NIR-II image-guided sentinel lymph node (SLN) surgery.

## Abstract 2

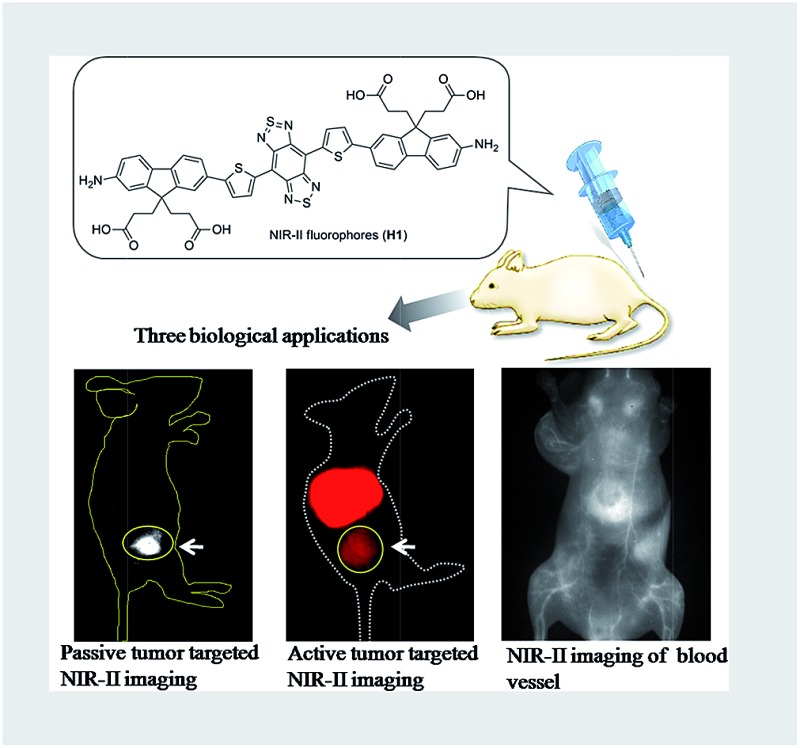
This work presents the establishment of novel bright-emission small-molecule NIR-II fluorophores for *in vivo* tumor imaging and NIR-II image-guided sentinel lymph node surgery.

## Introduction

As fluorescence imaging *in vivo* continues to gain increasing interest and expand within both academic and clinical settings, a transition shifted to longer wavelengths in the second near-infrared window (NIR-II, 1000–1700 nm) region could have clear-cut advantages of deeper tissue imaging, high spatial resolution, and high contrast owing to minimal auto-fluorescence and tissue scattering.^1,2^ Improved imaging quality, superior lymphatic imaging, deeper brain tumor imaging, and higher tumor-to-background ratios have been achieved recently compared to the conventional NIR-I region (750–900 nm).^3,4^ Developing novel NIR-II emitting agents for biomedical applications thus has high significance and directly promotes the field of biomedicine. Thus far, organic and inorganic materials such as small molecules,^5–7^ conjugated polymers,^8^ carbon nanotubes,^9,10^ quantum dots (QDs),^11–13^ and rare earth nanoparticles,^14,15^ have been actively employed for NIR-II fluorescence imaging.^16^ However, reports of NIR-II fluorophores are still scarce and the small-molecule fluorescent cores are relatively limited compared with their NIR-I counterparts.^6^ Hence, it prompts us to expand the library of small-molecule NIR-II fluorophores, which will significantly promote the widespread use of NIR-II imaging modality.

Several types of small-molecule NIR-II dyes with favorable excretion pharmacokinetics have been reported, in which the fluorophore units are generally composed of aromatic conjugate units based on a donor–acceptor–donor (D–A–D) structure with a benzobisthiadiazole (BBTD) core.^5,6^ Among them, a small-molecule probe **Q4** was selected as a scaffold for the facile construction of NIR-II agent SCH1100 for targeted prostate cancer imaging.^6,17^ However, the complexity and multiple synthetic steps with low yields, tedious chromatographic isolation and the weak brightness of **Q4** heavily hinder the wide application of such a promising agent in preclinical and clinical studies. Hence, many efforts should be made to simplify the synthetic strategy and optimize the brightness of small-molecule NIR-II fluorophores.

**Fig. 1:**
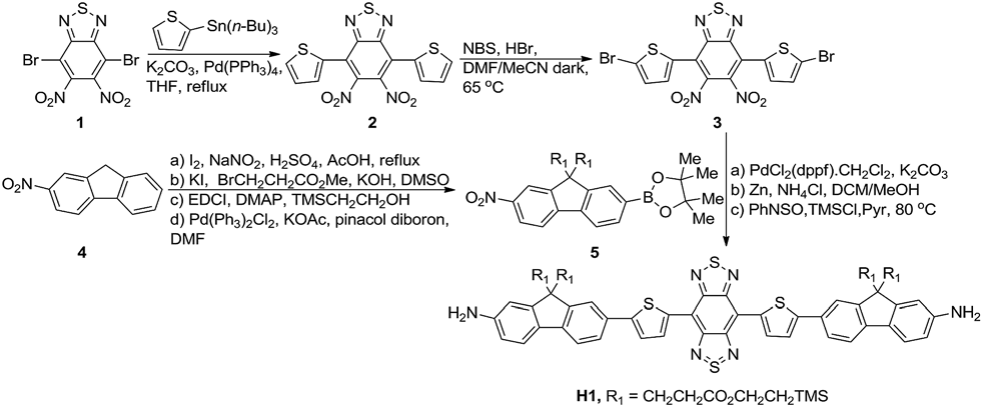
Facile synthesis of compound **H1**.

Herein, we report a novel small-molecule NIR-II dye **H1** with an improved synthetic protocol and fluorescence characteristics. At longer fluorescence emission wavelengths in the NIR-II region, the increased bandgap of molecular fluorophores generally gives way to reduce interactions between the conjugated backbone and other molecules, causing a high fluorescence quantum yield (QY).^18,19,20^ Therefore, in this work, R_1_ substituent groups on the sp^3^ carbon of the fluorene group are out-of-plane of the π-conjugated system and thus prevent intermolecular stacking that leads to fluorescence quenching. Meanwhile, newly introduced 2-amino 9,9-dialkyl-substituted fluorene moieties distort the BBTD backbone and thus effectively tune the electrostatic potential distribution and the bandgap to the desired range. Moreover, the fluorene moieties act as both the electron donor and protecting groups with the benefits of a compact molecular structure and shielding the backbone from aggregation (Fig. 1). Finally, three types of NIR-II probes (**SXH**, **SDH**, and **H1 NPs**) have been facilely prepared according to the **H1** scaffold, and demonstrated different biomedical applications such as passive/active tumor targeted imaging, high resolution imaging of blood vessels on tumors and the whole body, and image-guided sentinel lymph node surgery in the NIR-II imaging region. The novel organic fluorescent compound **H1** provides unprecedented opportunities for the construction of a variety of NIR-II probes for *in vivo* molecular imaging.

## Results and discussion

**Fig. 2:**
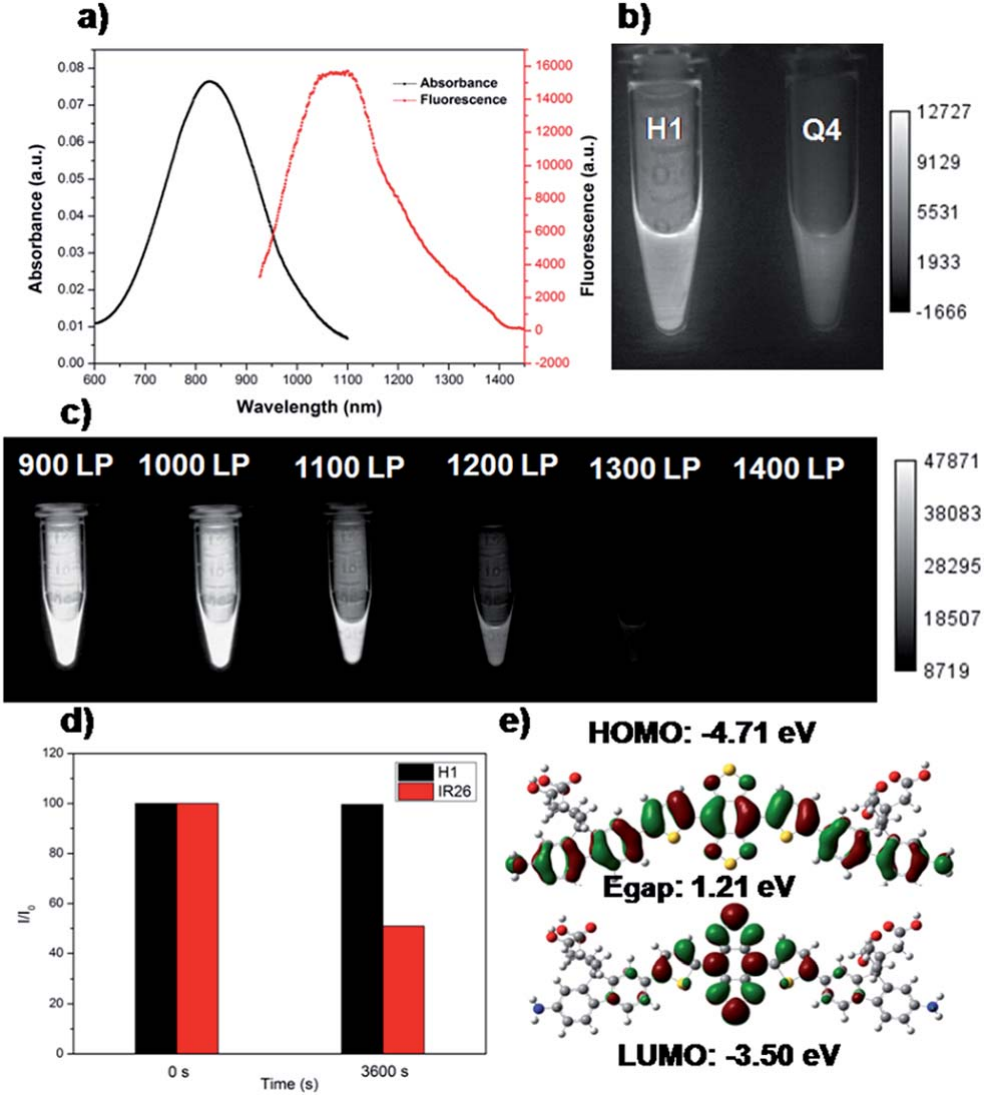
(a) UV absorbance of **H1** and NIR-II fluorescence emission of **H1** with a peak at ∼1100 nm under an 808 nm excitation laser (exposure time: 10 ms). (b) Comparison of NIR-II signals of **H1** and **Q4** under an 808 nm excitation laser (exposure time: 20 ms). (c) Comparison of NIR-II signals of **H1** under various long-pass (LP) filters (900–1400 nm). (d) Comparison of the photo-stability of **H1** and **IR-26** in dichloromethane under continuous 808 nm excitation for 1 h. (e) HOMO and LUMO orbital surfaces of **H1** using the DFT B3LYP/6-31G(d) scrf = (cpcm, solvent = CH_2_Cl_2_) method. *E*_gap_ = *E*_LUMO_ – *E*_HOMO_.

As shown in Fig. 1, compound **H1** was synthesized by a convergent route in 15% overall yield over 6 steps from commercially available starting materials without tedious chromatographic isolation (see ESI†). All compounds were characterized by NMR and ESI-MS, and also exhibited good solubility in common organic solvents such as CH_2_Cl_2_ and THF (see ESI†). By modulating the non-resonant side chains of the carboxylic acid groups, the final NIR-II fluorophores could be systematically altered to tune the hydrophobicity, polarity and efficient conjugation of bio-targets. The UV-vis-NIR absorption band of **H1** was at 600–1000 nm (in CH_2_Cl_2_) due to the formation of a strong charge-transfer structure between the D–A–D units (Fig. 2a). Meanwhile, the fluorescence emission spectrum of **H1** was obtained and demonstrated a peak emission wavelength at ∼1100 nm (Fig. 2a). The results indicated that the brightness of the fluorescence signals of **H1** was superior to that of **Q4** (Fig. 2b). Furthermore, the NIR-II signals of **H1** were investigated under various LP filters (900–1400 nm) and no signals were observed with the 1300 nm and 1400 nm filters (Fig. 2c). **H1** has exhibited high photo-stability compared to **IR-26**, with negligible decay under continuous excitation for 1 h (Fig. 2d). The calculated HOMO and LUMO orbital surfaces of **H1** have shown a larger band gap compared to that of **Q4**, leading to a higher performing fluorophore (Fig. 2e and Table S1†). The QY of **H1** was ∼2.0% under 785 nm excitation (in CH_2_Cl_2_, measured against an **IR-26** reference with a nominal quantum yield of 0.5%, Fig. S1†). All these data demonstrated **H1** could be a promising NIR-II dye, suitable for further NIR-II imaging applications.

**Fig. 3:**
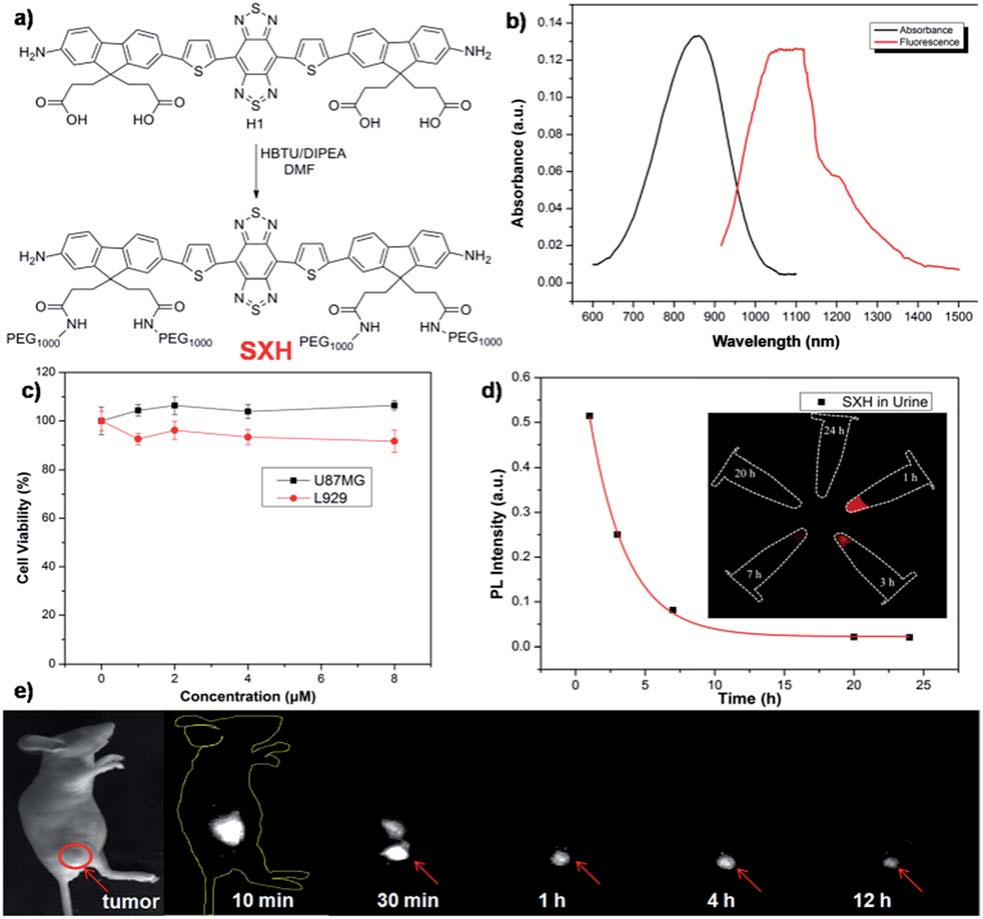
(a) A schematic design of **SXH** showing four carboxylic acid groups of **H1** conjugated with PEG_1000_ chains. (b) UV absorbance of **SXH** and NIR-II fluorescence emission of **SXH** with a peak at ∼1100 nm under an 808 nm excitation laser (solvents: water, exposure time: 10 ms). (c) Cellular toxicity of **SXH** with different doses (2, 4, 6, and 8 µM) in U87MG and L920 cells. (d) **SXH** agglomerated cumulative urine excretion curve during 24 h post-injection. (e) Non-specific targeting imaging of the U87MG tumor based on **SXH** under an 808 nm excitation (1000 LP and 200 ms).

**SXH** was easily prepared through conjugation of four carboxylic acid groups of **H1** with PEG_1000_ chains (Fig. 3a and ESI†). **SXH** was purified using HPLC and characterized using MALDI-TOF-MS (see ESI†). **SXH** exhibited high aqueous solubility and the fluorescence emission spectrum of **SXH** demonstrated a similar emission wavelength at ∼1100 nm to that of **H1** (Fig. 3b). The results from a cytotoxicity study further indicated the high viability of U87MG and L929 cells after 24 h of incubation with different doses of **SXH** (2, 4, 6, and 8 µM), demonstrating the high biocompatibility of **SXH** (Fig. 3c). Excretion kinetics were investigated by intravenous injection of 100 µg of **SXH** into U87MG tumor-bearing nude mice (*n* = 3) for glioblastoma (GBM) imaging and collecting urine during the course of 24 h post-injection (P.I.). Glioblastoma, the most common primary brain tumor in adults, is usually rapidly fatal.^21^ The care for patients with a newly diagnosed glioblastoma entails surgical resection and concurrent radiation therapy (RT) and chemotherapy. Pharmacokinetics of **SXH** demonstrated rapid urine excretion, with ∼90% of **SXH** removal through the renal system within the first few hours of the 24 h post-injection (Fig. 3d and Fig. S2†). Finally, U87MG tumor-bearing nude mice (*n* = 3) were injected with 100 µg of **SXH** and non-invasive NIR-II fluorescence imaging of the glioblastoma tumor was conducted at particular time points. After 30 min post-injection, the tumor was clearly visible with a T/NT ratio of ∼4 and showed passive uptake at all time points due to the non-specific diffusion and accumulation of **SXH** (Fig. 3e). *Ex vivo* biodistribution studies were further performed at 24 h post-injection of the probe to evaluate the distribution of **SXH** in major organs (Fig. S3†). It was found that **SXH** mainly accumulated in the kidneys, suggesting that the clearance route of **SXH** was through the renal system. In addition, a high level of accumulation was also observed in the tumor, indicating that **SXH** can passively target tumors and be used for future cancer theranostic applications (Fig. S3†).

**Fig. 4:**
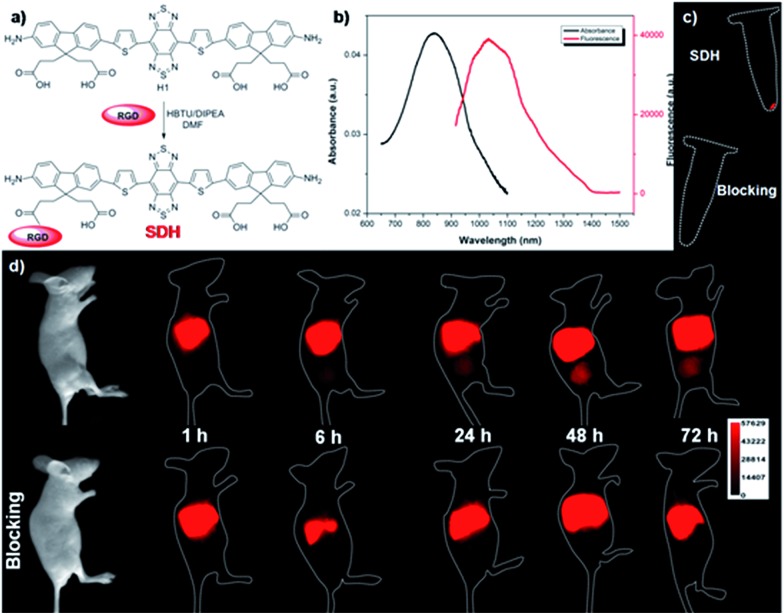
(a) A schematic of **SDH** showing one of the carboxylic acid groups of **H1** conjugated with targeted ligand RGD peptide. (b) UV absorbance and NIR-II fluorescence emission of **SDH**. (c) NIR-II signals of U87MG cell labelling by **SDH** and SDH + excess RGD as a blocking agent (block group) under 808 nm excitation (1000LP and 100 ms). (d) NIR-II images of U87MG tumor mice (*n* = 3) at different time points (1, 6, 24, 48, and 72 h) after tail vein injection of **SDH** with or without the blocking agent RGD (500 µg) under 808 nm excitation (1000LP and 200 ms).

Although PEGylation of **H1** provided a rapidly excreted, versatile contrast agent capable of passive tumor uptake, **H1** could provide more tumor-specific targeting by linking to a molecular imaging ligand. We next demonstrated the application of **H1** for receptor-targeted glioma imaging. Integrin α_V_β_3_ has high expression levels in several malignant diseases including glioblastoma and are established biomarkers for metastatic diseases.^22^ The integrin targeting peptide RGD (arginine–glycine–aspartic acid) has shown promising results for non-invasive molecular imaging of integrin α_V_β_3_ expression in the NIR-I region.^23^ Considering the advantages of NIR-II imaging, a novel integrin α_V_β_3_-targeted NIR-II fluorophore, **SDH**, was developed and explored to investigate its imaging properties *in vivo*. **SDH** was prepared through conjugation of **H1** with a mono-c(RGDfk) targeting peptide (Fig. 4a), and then purified by HPLC and characterized by MALDI-TOF [calcd for C_72_H_79_N_15_O_14_S_4_: 1589.481, found: *m*/*z* 1589.669]. The fluorescence emission spectrum of **SDH** demonstrates an emission wavelength at ∼1050 nm (Fig. 4b). The cell toxicity study also indicated the high biocompatibility of **SDH** *in vitro* (U87MG and L929 cells after 24 h incubation with 2, 4, 6, and 8 µM doses of **SDH**, Fig. S4†). These results demonstrated that **SDH**, as a promising and biocompatible NIR-II fluorescent probe, is suitable for tumor targeting imaging.

**SDH** was then intravenously injected (100 µg) into U87MG tumor-bearing mice (*n* = 3 per group). From NIR-II imaging data, the U87MG tumor could be clearly visualized from the surrounding background tissue during 24–72 h post-injection (P.I.) (Fig. 4d, 1000LP, 200 ms), and the tumor uptake reached a maximum at 48 h. The specificity of **SDH** for integrin α_V_β_3_ was confirmed by the blocking experiment. The tumors fluorescence signals were successfully reduced at all time points after co-injection of RGD peptide (500 µg) with **SDH** for NIR-II imaging (Fig. 4d). An *ex vivo* biodistribution study indicated that high accumulation was observed in the liver and kidneys, which suggested that the clearance routes of **SDH** were through both hepatobiliary and renal systems (Fig. S5†). In addition, the uptake of **SDH** in tumors was far beyond that of other normal organs and no uptake could be observed in the blocking group, which further confirmed the good integrin α_V_β_3_-targeted ability and specificity of **SDH** (Fig. S5 and S6†). Hence, the excellent translation ability of **SDH** represents a highly promising fluorescent probe for non-invasive monitoring of early stage glioblastoma in the NIR-II region.

**Fig. 5:**
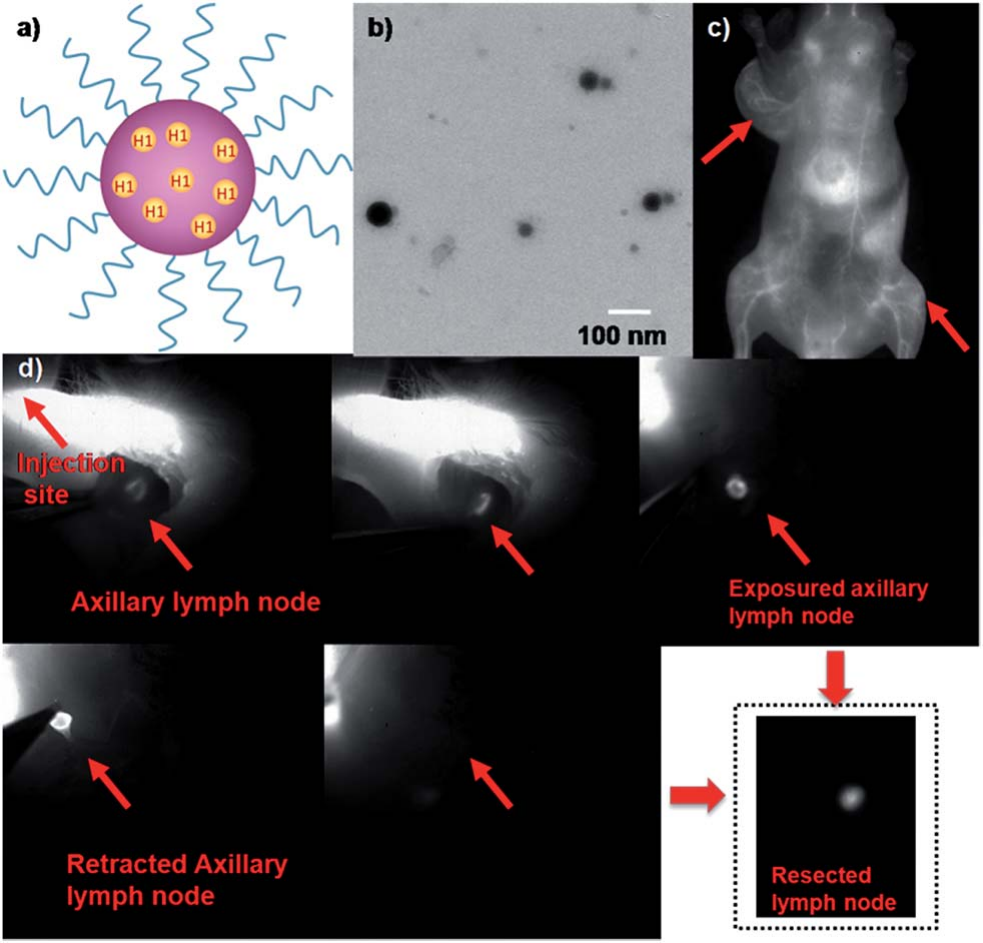
(a) A schematic of **H1 NPs** showing the **H1** core and a hydrophilic PEG shell. (b) The TEM image of **H1 NPs**. (c) The NIR-II image of the blood vessels of the whole body and U87MG tumors after tail vein injection of **H1 NPs** under 808 nm excitation, 1000 LP and 100 ms; red arrows indicate the tumor. (d) NIR-II imaging-guided sentinel lymph node surgery on the C57BL/6J model (1000LP and 200 ms).

An emerging fluorescence imaging application of NIR fluorophores, such as indocyanine green (ICG), is currently undergoing clinical trials in detecting sentinel lymph nodes (SLNs) for surgical resection.^24^ Selectively removing sentinel lymph nodes alleviates lymphedema and other ailments that would be caused by total lymph node removal, which is performed to prevent cancer metastasis. Recent advances in NIR-I fluorescence molecular imaging (FMI) for intraoperative image-guided cancer resection have introduced new frontiers for FMI-based therapeutic interventions in preclinical research and clinical applications. The inherent advantages of this novel technology compared to bright light surgery, such as its high sensitivity, high superficial resolution, low cost, and real-time imaging capacity, have stimulated the development of fluorescent probes with different molecular features.^25–33^ To demonstrate the feasibility of **H1** for SLN surgery, **H1** was encapsulated into a PEGylated surfactant, DSPE-mPEG_5000_, to prepare water-soluble and biocompatible NIR-II nanoprobes, **H1 NPs** (Fig. 5a). The prepared **H1 NPs** showed high monodispersity and homogeneity with an average particle size of ∼70.0 nm by transmission electron microscopy (TEM, Fig. 5b) and a hydrodynamic diameter of ∼80.0 nm as determined by dynamic light scattering (DLS, Fig. S7†). The fluorescence emission wavelength of **H1 NPs** was ∼1100 nm (Fig. S8†). The result of the cytotoxicity study indicated the high biocompatibility of **H1 NPs** (Fig. S9†). The amount of **H1** encapsulated in the liposomes was measured by a UV/VIS spectrophotometer at 874 nm (ESI†). The dye encapsulation efficiency of **H1 NPs** was 79.8 ± 0.6% (*n* = 3).

The U87MG tumor-bearing nude mice (*n* = 3) were injected with 150 µL of **H1 NPs**. Immediately after injection, the superior imaging of blood vessels of the whole body and tumor could be clearly observed from the surrounding background tissue using NIR-II imaging (Fig. 5c). Based on this promising result, we applied **H1 NPs** for lymph node imaging and image-guided surgery on the C57BL/6J model (Fig. 5d). C57BL/6J mice were injected with 100 µL of **H1 NPs**, with the help of NIR-II imaging, a SLN was successfully determined, even covered with soft tissue. After the SLN was exposed, it was then resected thoroughly. More importantly, the border of the SLN was easily distinguished, avoiding unnecessary damage of surrounding tissue such as nerves, vessels and tendons.

## Conclusions

In summary, we have developed a newly designed and facilely prepared NIR-II fluorophore **H1** with improved fluorescence by introducing 2-amino 9,9-dialkyl-substituted fluorene as a donor into the backbone. Based on this **H1** scaffold, three types of NIR-II imaging probe, **SXH**, **SDH** and **H1 NPs**, have been prepared and allow for integrin α_V_β_3_-targeted glioma imaging. To the best of our knowledge, this is the first time that a NIR-II molecular fluorophore has been shown to delineate tumours from surrounding normal tissue. Various biomedical applications such as high resolution imaging of blood vessels on tumours and the whole body of living mice using **H1 NPs** were also performed through a passive targeted probe. With the help of NIR-II imaging, an SLN was successfully determined and resected thoroughly. Future work will focus on performing intraoperative image-guided cancer surgery in orthotopic models and specialized pharmacokinetic studies with the eventual goal of initiating clinical trials with a NIR-II small-molecule fluorophore.
