# Supplementary material for: Novel bright-emission small-molecule NIR-II fluorophores for in vivo tumor imaging and image-guided surgery
Source: Chem Sci. 2017 Feb 21;8(5):3489–93. doi: 10.1039/c7sc00251c (PMC5418643; doi:10.1039/c7sc00251c)

Electronic Supplementary Material (ESI) for Chemical Science.
This journal is © The Royal Society of Chemistry 2017

## Supported Information

# Novel bright-emission small-molecule NIR-II fluorophores for *in vivo* tumor imaging and image-guided surgery

Yao Sun,[a,b]† Mingmin Ding,[a]† Xiaodong Zeng,[a]† Yuling Xiao,[a]† Huaping Wu,[a] Hui Zhou,[a] Bingbing Ding,[a] Chunrong Qu,[a] Wei Hou,[a] Er-BuAGA,[d] Yejun Zhang,[e] Zhen Cheng,[c] Xuechuan Hong*[a,d]

| Dye | HOMO Energy (eV) | LUMO Energy (eV) | $E_{gap}$ (eV) |
|---|---|---|---|
| H1 | 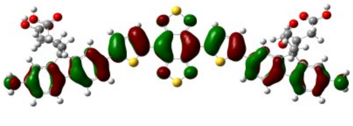 -4.71 | 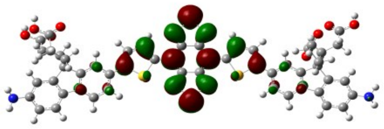 -3.50 | 1.21 |
| Q4 | 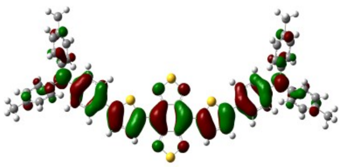 -4.58 | 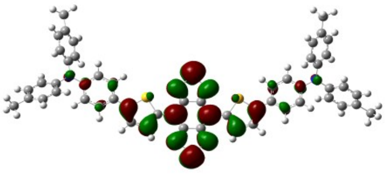 -3.46 | 1.12 |

**Table S1.** Comparison of HOMO and LUMO orbital surfaces of **H1** and **Q4** using DFT B3LYP/6-31G(d) scrf = (cpcm, solvent=dichloromethane) method. $E_{gap} = E_{LUMO}-E_{HOMO}$

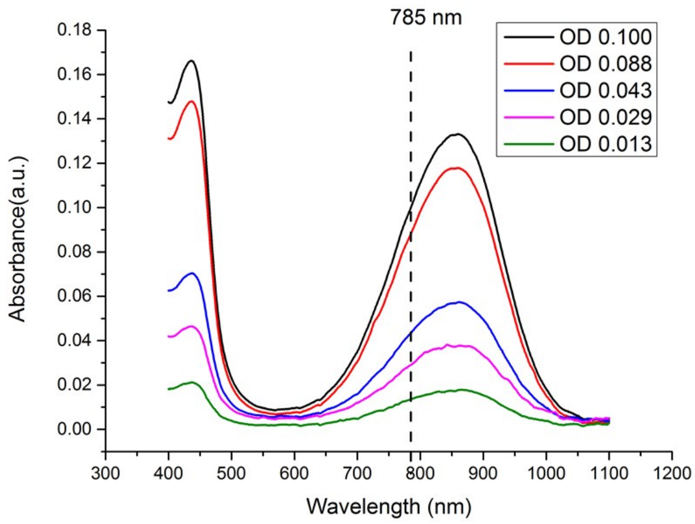

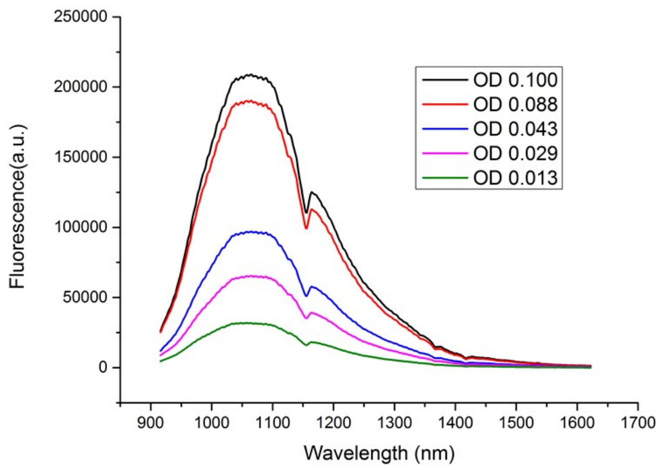


**Figure S1.** Quantum yield Measurements of **H1**. In order to measure the quantum yield of **H1**, a reference IR-26 (0.5%) was chosen.[1,2] Five difference concentrations were measured and the integrated fluorescence was plotted against absorbance for both IR-26 and **H1**. Comparison of the slopes led to the determination of the quantum yield of **H1**. The quantum yield was calculated in the following manner:

$$QYsample = QYref \times \frac{Slopesample}{Sloperef} \times \frac{nsample^2}{nref^2}$$

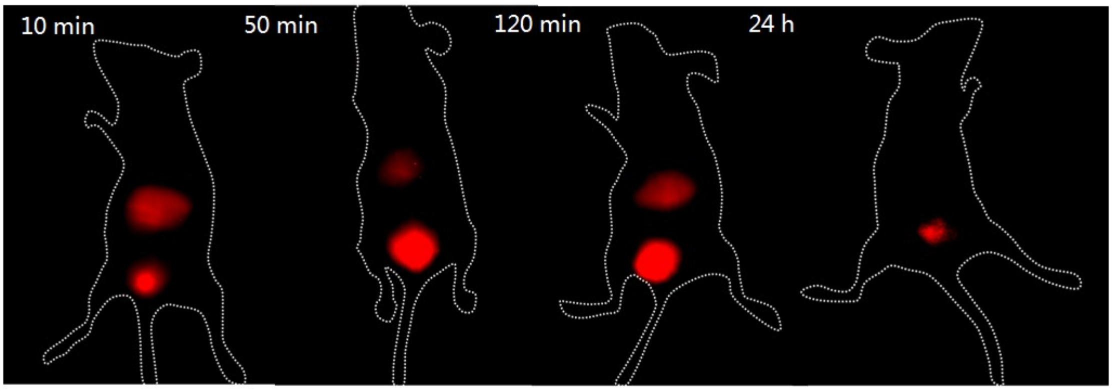


**Figure S2.** The **SXH** demonstrating renal excretion showing bladder fluorescent signals (n = 3 mice)

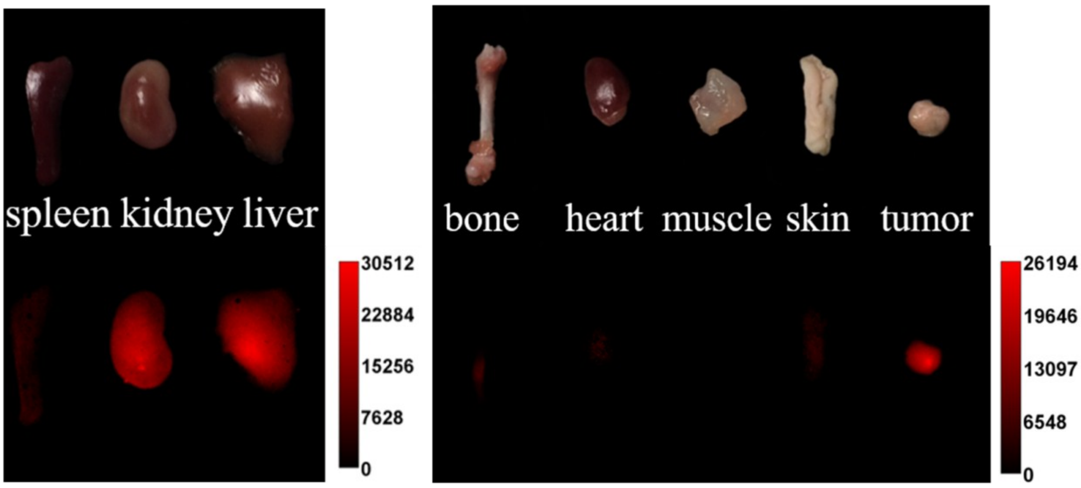


**Figure S3.** The biodistribution of **SXH** in tumor mice at 24 h under an 808 nm excitation (1000 LP and 200 ms)

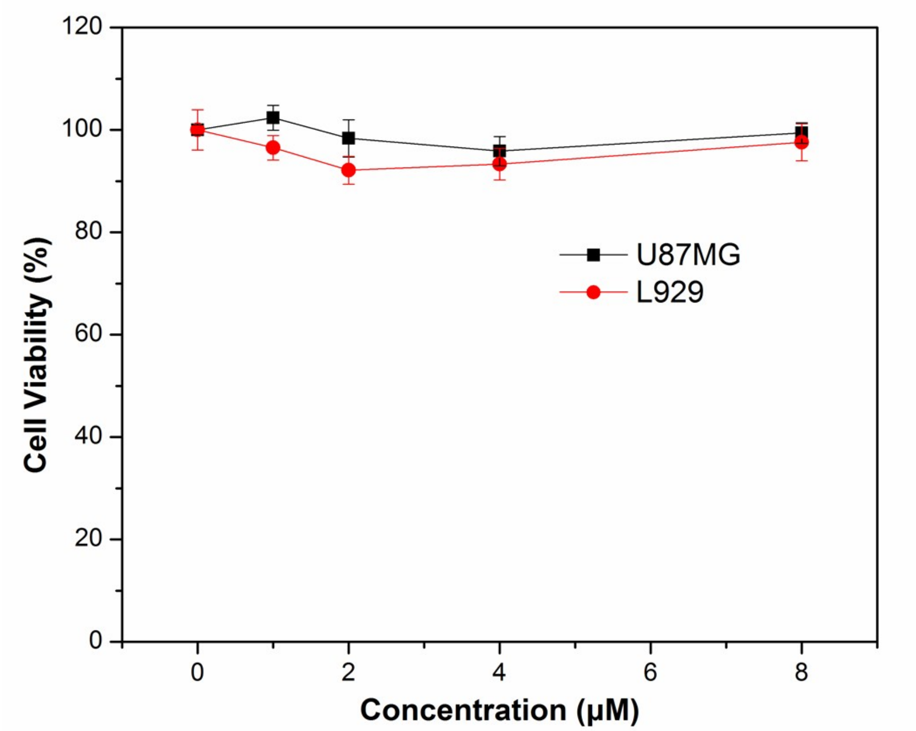


**Figure S4.** Cellular toxicity of **SDH**. Cell toxicity was assayed utilizing the U87MG and L929 cell lines

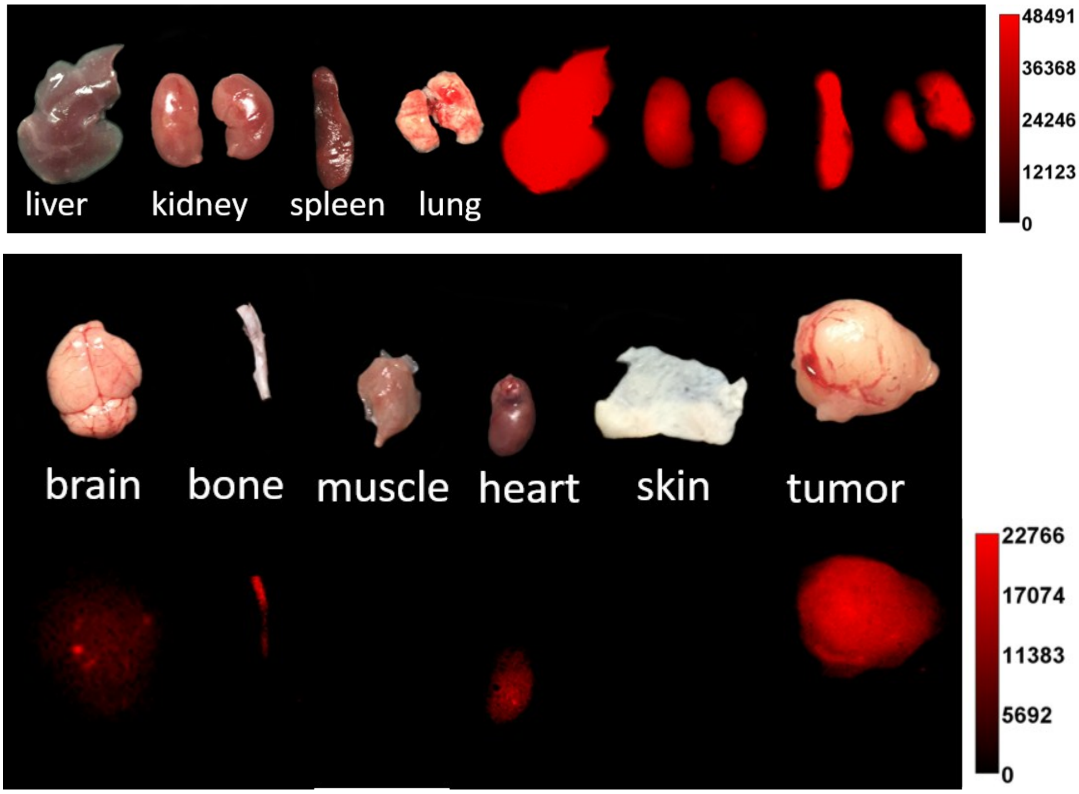

**Figure S5.** The biodistribution of **SDH** in tumor mice at 72 h under an 808 nm excitation (1000LP and 200 ms)

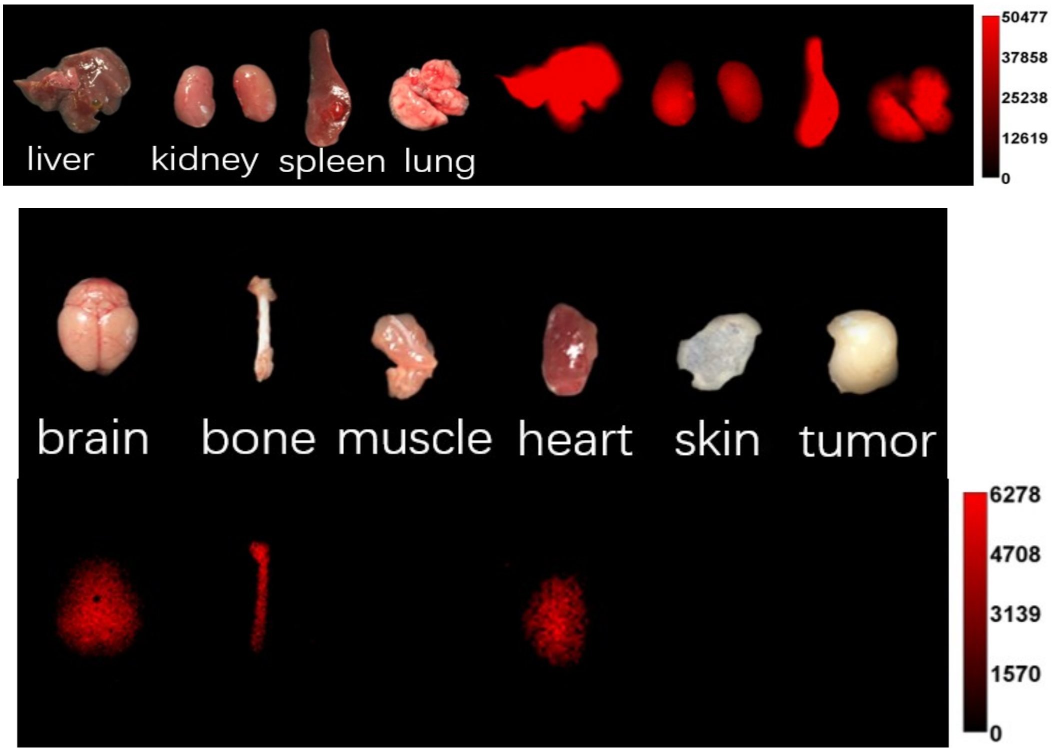

**Figure S6.** The biodistribution of **SDH** (with RGD blocking group) in tumor mice at 72 h under an 808 nm excitation (1000LP and 200 ms)

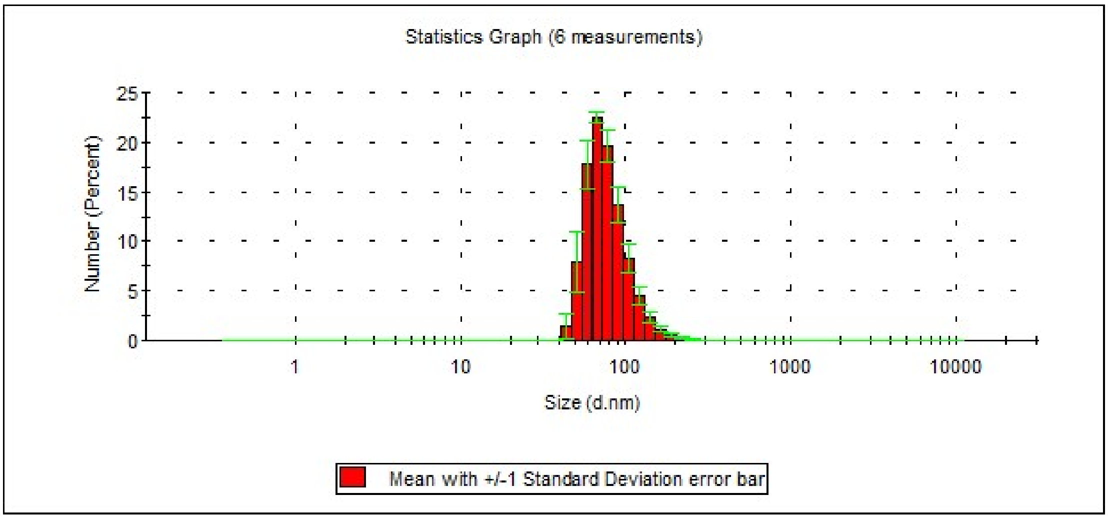


**Figure S7.** The size distribution of **H1 NPs** in water based on DLS measurement.

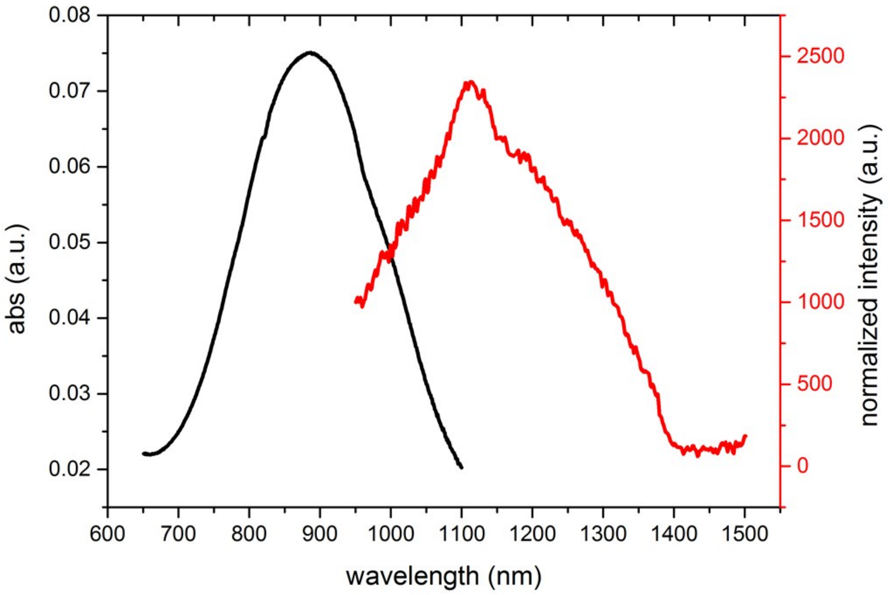


**Figure S8.** The emission and absorption of **H1 NPs** in water.

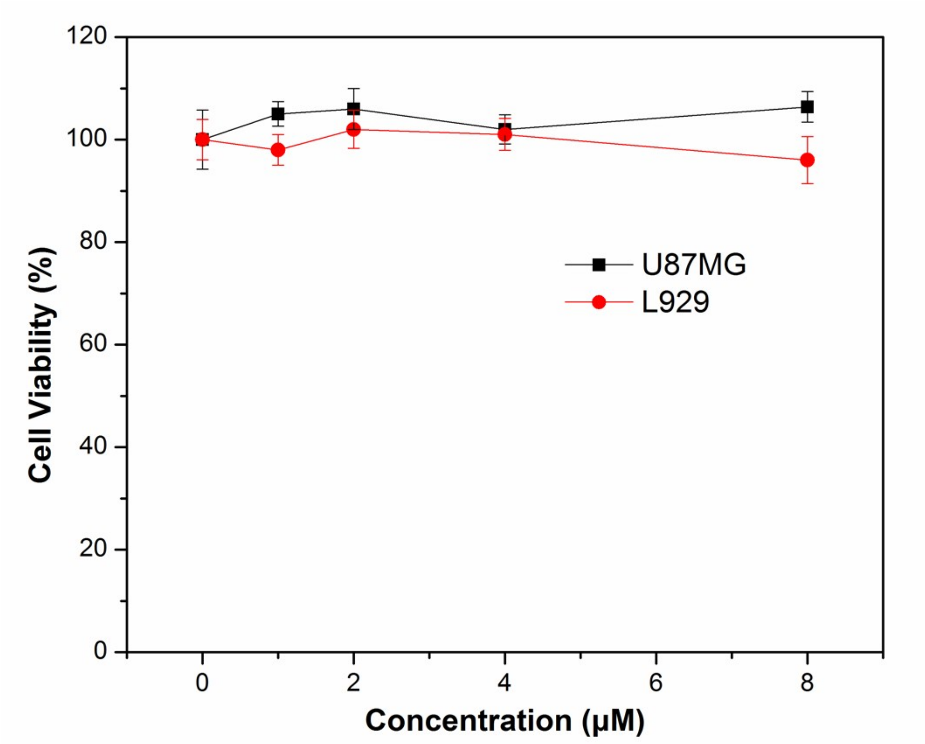


**Figure S9.** Cellular toxicity of **H1 NPs**. Cell toxicity was assayed utilizing the U87MG and L929 cell lines

## Materials and General Experimental Methods.

The $^{1}H$ and $^{13}C$ NMR spectra were acquired on a Bruker AV400 magnetic resonance spectrometer. Chemical shifts (ppm) were reported relative to internal $CDCl_3$ ($^{1}H$, 7.26 ppm and $^{13}C$, 77.0 ppm) and DMSO-$d_6$ ($^{1}H$, 2.49 ppm and $^{13}C$, 39.0 ppm). High-resolution mass spectra were performed on Thermo Scientific Q Exactive Focus mass spectrometer. MALDI-TOF-MS experiments were performed on an Applied Biosystems 4700 MALDI TOF mass spectrometer. UV-Vis absorbance of the probe was recorded on a PerkinElmer Lambda 25 UV-Vis spectrophotometer. The NIR-II system was purchased from Suzhou NIR-Optics Technologies CO., Ltd. NIR fluorescence spectrum was recorded on an Applied NanoFluorescence spectrometer at room temperature with an excitation laser source of 785 nm. Hydrodynamic diameter was measured using a Malvern Zetasizer Nano ZS. Transmission electron microscopy (TEM) images were recorded on a Hitachi TEM system at an accelerating voltage of 100 kV. Preparative high performance liquid chromatography (HPLC) was performed on a Dionex HPLC System with UV-Vis detection A reversed-phase C4 (Ultimate, 5 μm, 4.6 × 250 mm) column was used for semi-preparation (mobile phase: water/acetonitrile with 0.06 % TFA). Tetrahydrofuran (THF)was freshly distilled from sodium/benzophenone. *N,N*-Dimethylformamide (DMF) and dichloromethane($CH_2Cl_2$)were distilled from calcium hydride. $PEG_{1000}$-$NH_2$was purchased from Peng Sheng Biological. All other standard synthesis reagents were purchased from commercial suppliers (such as Aldrich, Energy Chemical) and used without further purification unless otherwise noted. TLC analysis was performed on silica gel plates and column chromatography was conducted over silica gel (mesh 200-300), both of which were obtained from the Qingdao Ocean Chemicals.

## Synthesis and characterization

## Synthesis of H1

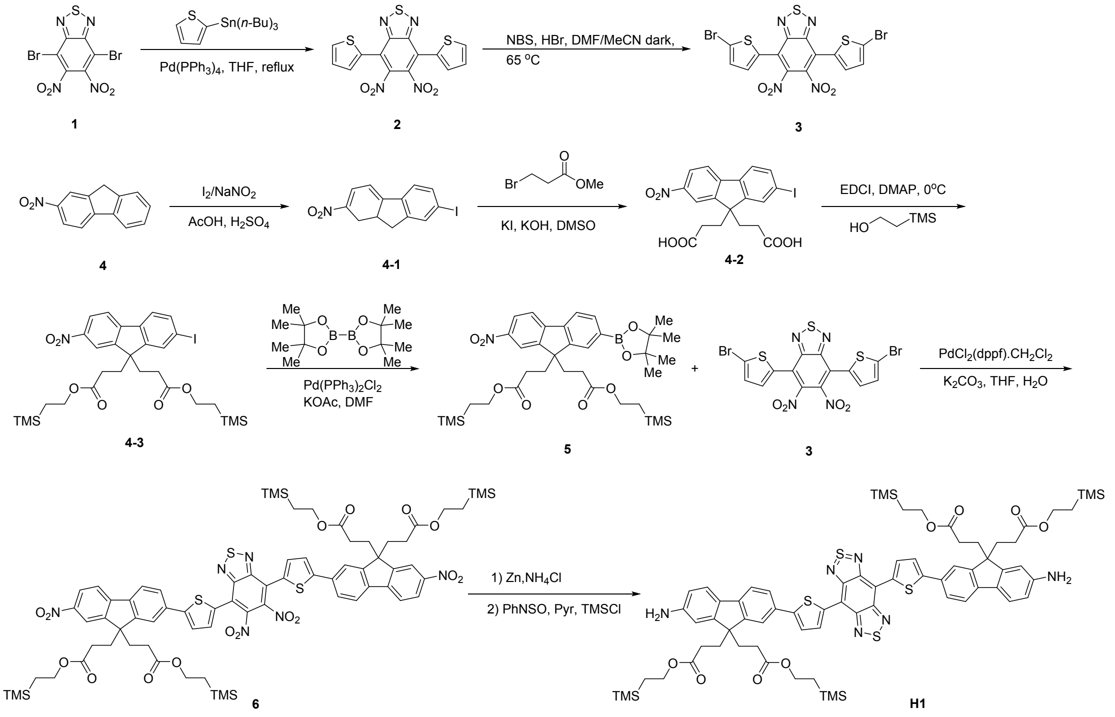

**Synthesis of compound 2**

This compound was prepared by a modified procedure according to previous reports.[3] To a solution of 4,7-dibromo-5,6-dinitro-benzo[1,2,5]thiadiazole (1.92 g, 5 mmol) and 2-thienyl tributylstannane (4.66 g, 12.5 mmol) in freshly distilled THF (35 mL) was bubbled with argon for 10 min. $Pd(PPh_3)_4$ (289 mg, 0.25 mmol) was added to the above reaction mixture under an argon atmosphere. The mixture was heated to reflux and it was stirring overnight. After cooling, saturated aqueous potassium fluoride (40 mL) was added and stirred at room temperature for 1 hour. The reaction mixture was filtered through Celite pad, the filter cake was washed with dichloromethane. The organic layer was washed with saturated aqueous brine, dried over anhydrous magnesium sulfate and the solvent was concentration under reduced pressure. The crude product was recrystallized from ethyl acetate. The desired product **2** (1.8 g, 92% yield) was obtained as an orange powder.

$^1H$ NMR (400 MHz, $CDCl_3$) δ 7.74 (dd, $J$ = 5.1, 0.9 Hz, 2H), 7.52 (dd, $J$ = 3.7, 0.9 Hz, 2H), 7.24 (dd, $J$ = 5.0, 3.8 Hz, 2H).

**Synthesis of compound 3**

This compound was prepared by a modified procedure according to previous reports.[4] 5,6-dinitro-4,7-di-thiophen-2-yl-benzo[1,2,5]thiadiazole (780 mg, 2 mmol) was dissolved in a mixture of DMF (10 mL) and MeCN (5 mL). The solution was heated to 65°C and *N*-bromosuccinimide (783 mg, 4.4 mmol) was added in one portion and the reaction stirred in the dark. Then HBr (four drops, 48 weight % aqueous) was added to the reaction. After 3 h, thin layer chromatography analysis revealed three spots. Another portion of *N*-bromosuccinimide (480 mg, 2.7 mmol) was added and the reaction was allowed to continue for additional 2 h. When a total amount of 1263 mg *N*-bromosuccinimide (7.1 mmol) had been added, thin layer chromatography analysis indicated the complete conversion of the starting materials. After cooling, the reaction mixture was acidified with hydrochloric acid (180 mL, 2 M) and stirred at room temperature for 2 h. The precipitate was collected by filtration and washed with water and methanol to give the desired compound **3** (986 mg, 90%) as an orange red powder.

$^1H$ NMR (400 MHz, $CDCl_3$) δ 7.26 (d, $J$ = 3.8 Hz, 2H), 7.19 (d, $J$ = 4.0 Hz, 2H).

**Synthesis of compound 7-Iodo-2-nitrofluorene (4-1)**

This compound was prepared by a modified procedure according to previous reports.[5] To 2-nitrofluorene (**4**) (11.62 g, 55 mmol) in 350 mL of glacial acetic acid was added iodine (6.98 g, 27.5 mmol). The solution was stirred at room temperature for 15 min, and then concentrated $H_2SO_4$ (36 mL) and $NaNO_2$(3.795 g, 55 mmol) were added subsequently. The mixture was heated under reflux for 60 min. After cooling to room temperature, the solvent was quenched by 375 g of ice, and the yellow solid that formed was collected by filtration. Recrystallization from glacial acetic acid (65 mL) afforded 7-Iodo-2-nitrofluorene (17.4 g, 94%).

$^1H$ NMR (400 MHz, $CDCl_3$) δ 8.40 (s, 1H), 8.30 (d, *J* = 8.4 Hz, 1H), 7.99 (s, 1H), 7.85 (d, *J* = 8.5 Hz, 1H), 7.79 (d, *J* = 8.1 Hz, 1H), 7.61 (d, *J* = 8.0 Hz, 1H), 4.04 – 3.95 (m, 2H).

**Synthesis of compound 4-2**

7-Iodo-2-nitrofluorene (**4-1**) (6.07 g, 18 mmol) and potassium iodide (299 mg, 1.8 mmol) were dissolved in dimethyl sulfoxide (41 mL) under an argon atmosphere. Methyl 3-bromopropionate (4.32 mL, 39.6 mmol, 6.613 g) was added to the reaction mixture. Potassium hydroxide (5.05 g, 90 mmol) was added in 10 portions to the solution. The green reaction mixture was stirred at room temperature for 24 h and quenched with water. The mixture was acidified to pH 5 with 2 M aq. HCl solution, extracted with EtOAc (3×150 mL). The combined organic layers were dried with anhydrous magnesium sulfate, filtered and concentrated. Compound **4-2** (8.02 g, 92%) was obtained as a yellow solid which can be used in the next step without further purification.

$^1H$ NMR (400 MHz, DMSO) δ 11.98 (s, 2H), 8.43 (d, *J* = 2.0 Hz, 1H), 8.28 (dd, *J* = 8.4, 2.1 Hz, 1H), 8.15 – 8.06 (m, 2H), 7.84 (s, 2H), 2.48 – 2.27 (m, 4H), 1.31 (dd, *J* = 17.1, 9.9 Hz, 4H).
$^{13}C$ NMR (101 MHz, DMSO) δ 174.1, 152.6, 149.24, 147. 8, 146.8, 138.5, 137.4, 133.0, 124.4, 124.2, 121.5, 119.6, 97.3, 54.8, 33.69, 29.1.
HRMS (ESI) Calcd for: $C_{19}H_{16}INNaO_6^+$ ($[M+Na]^+$): 503.9920, found: 503.9913.

**Synthesis of compound 4-3**

To a solution of compound **4-2** (4.81 g, 10 mmol) and 2-trimethylsilylethanol (7.75 mL, 54 mmol, 6.39 g) in $CH_2Cl_2$ (24 mL) and DMF (36 mL) was cooled at -15 °C under an argon atmosphere. EDCI (9.59 g, 50 mmol) and DMAP (1.96 g, 16 mmol) were added in a single portion and the reaction mixture was stirred at -15 °C for 5 h. The reaction mixture was then allowed to warm at ambient temperature. After dilution with dichloromethane (300 mL), the organic layer was washed with saturated $NH_4Cl$ solution (200 mL), water (4×200 mL), saturated aqueous brine (100 mL), dried over anhydrous magnesium sulfate, filtered and concentrated to dryness. The residue was crystalized by *n*-hexane/EtOAc to yield compound **4-3** (4.74 g, 69%).

$^1H$ NMR (400 MHz, $CDCl_3$) δ 8.29 (dd, *J* = 8.4, 2.0 Hz, 1H), 8.22 (dd, *J* = 6.8, 1.9 Hz, 1H), 7.84 – 7.73 (m, 3H), 7.53 (d, *J* = 8.2 Hz, 1H), 4.01 – 3.91 (m, 4H), 2.51 – 2.38 (m, 4H), 1.58 – 1.46 (m, 4H), 0.88 – 0.78 (m, 4H), -0.03 (s, 18H).
$^{13}C$ NMR (101 MHz, DMSO) δ 172.5, 151.9, 148.9, 147.7, 147.0, 138.7, 137.5, 133.3, 124.4, 124.2, 121.6, 119.7, 97.2, 62.2, 55.0, 33.4, 29.3, 17.1, -1.1.
HRMS (ESI) Calcd for: $C_{29}H_{40}INNaO_6Si_2^+$ ($[M+Na]^+$): 704.1337, found: 704.1323.

**Synthesis of compound 5**

To a solution of compound **4-3** (2.045 g, 3 mmol), bis(pinacolate)diboron (0.914 g,

3.6 mmol) and KOAc (0.706 g, 7.2 mmol) and in DMF (60 mL) was added bis(triphenylphosphine)palladium(II) dichloride (210 mg, 0.3 mmol) under an argon atmosphere. Then the reaction mixture was heated in an oil bath at 80 °C for 2 h. After the reaction mixture was cooled down to room temperature, 60 mL ethyl acetate was added and solid was removed by filtration. The solution was dilute with water (120 mL) and extracted with *n*-hexane (3×60 mL). The combined organic layers were washed with water (4×60 mL), saturated aqueous brine (150 mL) and dried with anhydrous $MgSO_4$. The solvents were concentrated to 4 mL and cooled to -20 $^oC$ to give 1.11 g yellow solid **5** (yield: 54%).

$^1H$ NMR (400 MHz, $CDCl_3$) δ 8.29 (dd, *J* = 8.4, 2.0 Hz, 1H), 8.23 (d, *J* = 1.9 Hz, 1H), 7.89 – 7.77 (m, 4H), 3.95 (ddd, *J* = 8.2, 6.5, 3.0 Hz, 4H), 2.49 (t, *J* = 8.1 Hz, 4H), 1.54 (dd, *J* = 9.8, 6.5 Hz, 2H), 1.46 (dd, *J* = 10.1, 6.4 Hz, 2H), 1.39 (s, 12H), 0.86 – 0.79 (m, 4H), -0.03 (s, 18H).
$^{13}C$ NMR (101 MHz, $CDCl_3$) δ 174.5, 151.2, 150.0, 149.2, 148.9, 143.0, 136.5, 130.8, 125.6, 122.4, 122.3, 120.2, 85.7, 85.1, 64.2, 55.7, 35.9, 30.7, 26.6, 26.5, 18.7, 0.0.
HRMS (ESI) Calcd for: $C_{35}H_{52}BNNaO_8Si_2^+$ ($[M+Na]^+$): 704.3222, found: 704.3212.

**Synthesis of compound 6**
To a solution of compound **5** (70 mg, 0.1 mmol) and compound **3** (27.4 mg, 0.05 mmol) in THF (2.5 mL) was bubbled with argon for 5 min. Potassium carbonate (18 mg, 0.125 mmol) in 0.5 mL distilled water and 1,1'-Bis(diphenylphosphino)ferrocene-palladium(II)dichloride dichloromethane complex (9 mg, 0.01 mmol) were added to the above reaction mixture under an argon atmosphere. The mixture was heated in an oil bath at 75 °C for 14 h. After cooling to room temperature, the solvent was removed *in vacuo*. The residue was dissolved in dichloromethane, and the resulting solution was washed with water, saturated aqueous brine. After drying over anhydrous magnesium sulfate and removal of the solvents under reduced pressure, product **6** (67 mg, 90% yield) was obtained as a dark red solid which can be used in the next step without further purification.

$^1H$ NMR (400 MHz, $CDCl_3$) δ 8.37 (dd, *J* = 8.4, 1.7 Hz, 2H), 8.31 (s, 2H), 7.92 (t, *J* = 7.5 Hz, 4H), 7.83 (d, *J* = 8.3 Hz, 2H), 7.76 (s, 2H), 7.60 (dd, *J* = 9.9, 3.9 Hz, 4H), 4.00 (dd, *J* = 9.6, 7.5 Hz, 8H), 2.65 – 2.54 (m, 8H), 1.63 (dd, *J* = 13.8, 7.3 Hz, 8H), 0.89 – 0.84 (m, 8H), 0.00 (s, 36H).
$^{13}C$ NMR (101 MHz, $CDCl_3$) δ 174.3, 153.6, 152.0, 151.7, 151.0, 149.2, 148.3, 143.1, 141.0, 136.2, 133.8, 131.2, 128.2, 126.6, 125.9, 123.9, 122.5, 122.2, 122.1, 120.2, 64.4, 56.1, 36.07, 30.7, 18.8, 0.0.
MALDI-TOF-MS Calcd for: $C_{72}H_{85}N_6O_{16}S_3Si_4^+$ ($[M+H]^+$): 1497.4256, found: 1497.4619.

**Synthesis of compound H1**
Zinc dust (392 mg, 6 mmol) and $NH_4Cl$ (96.3 mg, 1.8 mmol) were added to a stirred solution of compound **6** (72 mg, 0.05 mmol) in dichloromethane (7.2 mL) and 90%

methanol (11.4 mL) under an argon atmosphere. After being stirred at room temperature for 4 h, the solution was filtered through Celite pad, diluted with dichloromethane, and washed with water, saturated aqueous $NaHCO_3$, and saturated aqueous brine. The organic phase was dried over anhydrous $MgSO_4$, filtered and concentrated under vacuum to afford a yellow solid which was utilized for the next step without further purification.
To a dark yellow solution in anhydrous pyridine (1 mL) was added *N*-thionylaniline (0.2 mL, 1.8 mmol, 247 mg) and chlorotrimethylsilane (0.3 mL, 3.5 mmol, 377 mg). The mixture was heated in an oil bath at 80°C for 20 h. The reaction mixture was allowed to cool down to room temperature, poured into iced water, extracted with dichloromethane. The combined organic layer was washed with water, saturated aqueous brine, dried over anhydrous $MgSO_4$, and concentrated *in vacuo*. The residue was purified by flash column chromatography on silica gel (10:1 petroleum ether: ethyl acetate to 100 :1 dichloromethane : methanol) to yield the product **H1** as a light dark yellow solid (34 mg, two step 48% yield).

$^1$H NMR (400 MHz, $CDCl_3$) δ 8.95 (d, *J* = 2.5 Hz, 2H), 7.74 (s, 2H), 7.68 (s, 2H), 7.56 (s, 2H), 7.50 (d, *J* = 8.0 Hz, 4H), 6.69 (d, *J* = 7.7 Hz, 4H), 4.03 – 3.97 (m, 8H), 2.58 – 2.45 (m, 4H), 2.45 – 2.35 (m, 4H), 1.69 (dd, *J* = 10.1, 5.3 Hz, 8H), 0.87 – 0.81 (m, 8H), -0.05 (s, 36H).
$^{13}$C NMR (101 MHz, $CDCl_3$) δ 175.3, 152.7, 151.6, 151.2, 149.2, 148.4, 143.6, 138.7, 135.6 133.4, 133.4, 127.2, 125.5, 122.8, 121.4, 120.7, 116.4, 111.1, 64.1, 55.1, 36.5, 30.8, 18.8, -0.0.
MALDI-TOF-MS Calcd for: $C_{72}H_{88}N_6O_8S_4Si_4^+$ ($[M]^+$): 1404.4623, found: 1404.0483.

**Synthesis of compound 7**

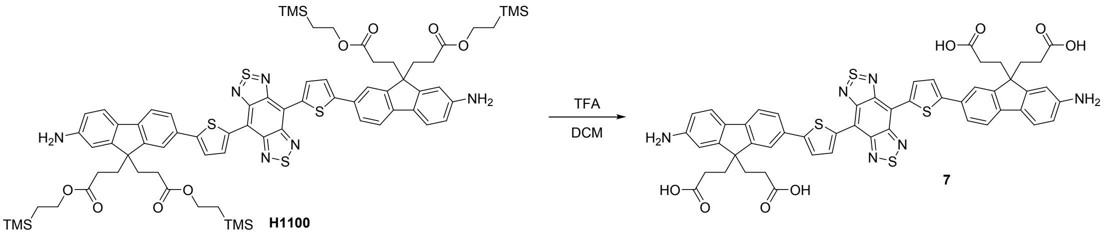


To a solution of compound **H1** (34 mg, 0.024 mmol) in DCM (3 mL) was cooled at 0 °C. Trifluoroacetic acid (3 mL) was added and the reaction mixture was stirred at 0 °C for 3 hours. The reaction mixture was slowly warmed to ambient temperature. The solvent was removed *in vacuo* and the crude product was washed by dichloromethane to yield the desired compound **7** as a dark solid which was used for the next step without further purification.
MALDI-TOF-MS Calcd for: $C_{52}H_{40}N_6O_8S_4^+$ ($[M]^+$): 1004.179, found:1004.301

**Synthesis of SXH**

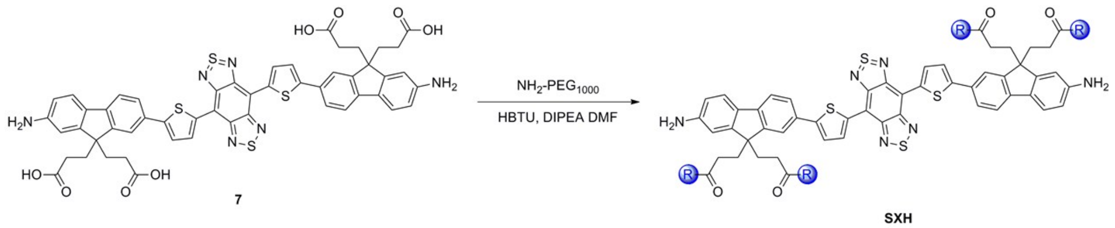


R = NH-PEG1000

To a solution of compound **7** (2 mg, 0.002 mmol) and $NH_2$-PEG (32 mg, 0.032 mmol, M.W. ~1000) in anhydrous DMF (1 mL) was added HBTU (15 mg, 0.04 mmol) and DIPEA (20 μL) at room temperature under an argon atmosphere. The reaction mixture was stirred at room temperature for 24 h. The crude product was precipitated in cold diethyl ether. The crude product was dissolved in water and purified by HPLC. Lyophilization of the purified material gave the desired product (3.2 mg, 33%) as a dark yellow solid. The final product **SXH** was confirmed using MALDI-TOF-MS. Expected M.W. ~4930, Measured M.W. ~4921.

**Synthesis of SDH**

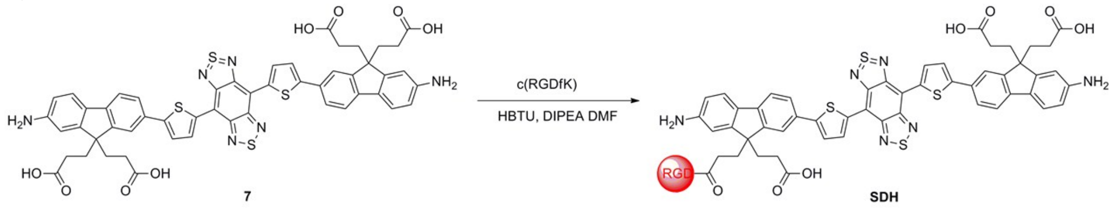


RGD = *c*(RGDfK) = *cyclo* (Arg-Gly-Asp-d-Phe-Lys)

To a solution of compound **7** (4 mg, 0.004 mmol) and *c*(RGDfK) (2.4 mg, 0.004 mmol) in anhydrous DMF (1 mL) was added HBTU (3 mg, 0.008 mmol) and DIPEA (10 μL) at room temperature under an argon atmosphere. The reaction mixture was stirred at room temperature for 24 h. The crude product was precipitated in cold $Et_2O$ and washed with $Et_2O$ three times. The crude product was dissolved in water/$CH_3CN$ (70/30) and purified by prep-HPLC. Lyophilization of the purified material gave the desired product **SDH** (1.4 mg, 22%) as a dark solid. MALDI-TOF-MS Calcd for: $C_{72}H_{79}N_{15}O_{14}S_4$: 1589.4814, found:1589.6691.

**Synthesis of H1 NPs**

The **H1** was dissolved in THF at a concentration of 0.1 mg/mL and mixed with an aqueous solution of DSPE-mPEG (5 kDa) at a concentration of 1 mg/mL with 1 : 9 volume ratio was stirred at room temperature for 2 h. After THF was removed by nitrogen flow, this reaction mixture was centrifuged at 12,000 rpm for 3 min using 30 kDa molecular weight cut-off filter to remove unreacted reagents, and washed by water 6 times using the same filter. The complex was finally re-dissolved in 0.5 mL water, followed by ultracentrifugation (50,000 rpm for 30 min). Supernatant was thus acquired as the solution of **H1NPs**. The amount of **H1** encapsulated in the liposomes was measured by UV/VIS spectrophotometer at 874 nm. The calibration curve was linear in the range of 2.5–40 μg/mL with a correlation coefficient of $R^2$ = 0.99918.

The encapsulation efficiency was defined as the ratio between the amount of **H1**encapsulated in the liposomes and that added in the liposomes preparation process. The dye encapsulation efficiency of **H1 NPs** was 79.8%±0.6%( *n* = 3).

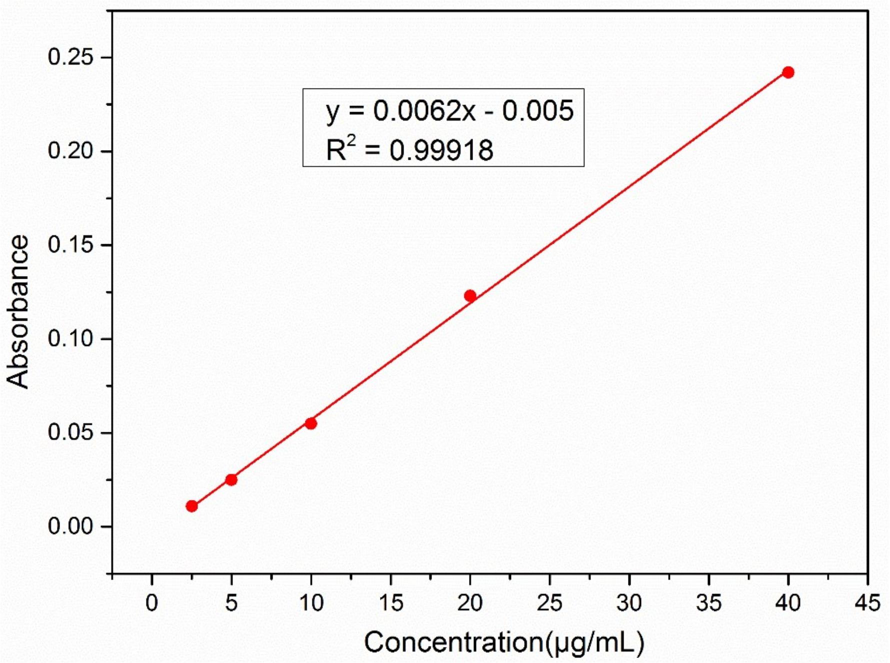


**Figure S10.** The calibration curve of **H1 NPs**

**Cell line and animal model.**

Human primary glioblastoma cell line U87MG and mouse fibroblastic cell line L929 were obtained from the American Type Culture Collection (ATCC). U87MG Cells were cultured in Low Glucose Dulbecco's Modified Eagle Medium (DMEM) medium (Gibco) supplemented with 10% fetal bovine serum, 100 IU/mL penicillin, 100 μg/mL streptomycin. L929 cells were cultured in Mimumum Essentiul Medium (MEM) medium (Gibco) supplemented with 10% fetal bovine serum, 100 IU/mL penicillin, 100 μg/mL streptomycin. All cells were cultured at 37 °C in a humidified with 5% $CO_2$ atmosphere. The U87MG rat tumor model was established by subcutaneous injection of U87MG cells (roughly $2 \times 10^6$ in 50 μL of Low Glucose DMEM medium) into the right leg of the 6-week-old female Balb/c nude mice (Suzhou Belda Bio-Pharmaceutical Co.). The tumor was allowed to reach ~5-10 mm in diameter (about 2 weeks after inoculation) and the mice were subjected to imaging studies. All animal experiments were performed according to the Chinese Regulations for the Administration of Affairs Concerning Experimental Animals.

**Cell cytotoxicity in MTT assay.**

In vitro cytotoxicity studies of **SXH** or **SDH** or **H1 NPs** on L929 cells and U87MG cells were performed by using a MTT cytotoxicity assay. Cells were cultured overnight in 96-well plates at $5\times10^3$ cells per well to allow cell attachment. The cells were incubated with 100 μL of fresh cell media containing of **SXH** or **SDH** or **H1 NPs** for 48 h. The final concentrations of **SXH** or **SDH** or **H1 NPs** in the culture

medium were fixed at 0, 1, 2, 4, and 8 μM in the experiment. Then the MTT (0.5 mg/mL) reagent (10 μL per well) was added for 4 h at 37 °C and DMSO (150 μL per well) was further incubated with cells to dissolve the precipitated formazan violet crystals at 37 °C for 15 min. The absorbance was measured at 490 nm by Perkin Elmer VICTOR X4. The following formula was used to calculate the viability of cell growth: Cell viability (%) = (mean of Absorbance value of treatment group /mean of Absorbance value of control) × 100.

**In vivo NIR-II fluorescence imaging of tumors.**

A 200 μL portion of **SXH** or **SDH** or **H1 NPs** at a 0.5 mg/mL concentration was injected intravenously into nude mice. During injection and imaging, the mice were anesthesized using a 2 L/min oxygen flow with 2% Isoflurane. NIR-II fluorescence images were collected using a two-dimensional InGaAs array (Suzhou Optics) for collecting photons in NIR-II. The excitation light was provided by an 808 nm diode laser. The emitted light from the animal was filtered through a 1000 nm long-pass filter coupled with the InGaAs camera for NIR-II imaging. The exposure time for all images was 200 ms.

***Ex vivo* biodistribution analysis.**

*Ex vivo* fluorescence imaging of organs and tissues, was performed with a home-built NIR-II fluorescence imaging system with an InGaAs camera under illumination of an 808 nm laser diode at a power density of roughly 50 $mW/cm^2$ and an exposure time of 200 ms. 72 h after injection of **SDH** or 24 h after injection of **SXH**, U87MG mice (n = 3 per group) were sacrificed, the major organs and tissues were collected. Then the NIR-II fluorescent signal of each subject was collected.

**Determination of the fluorescence quantum yield.**

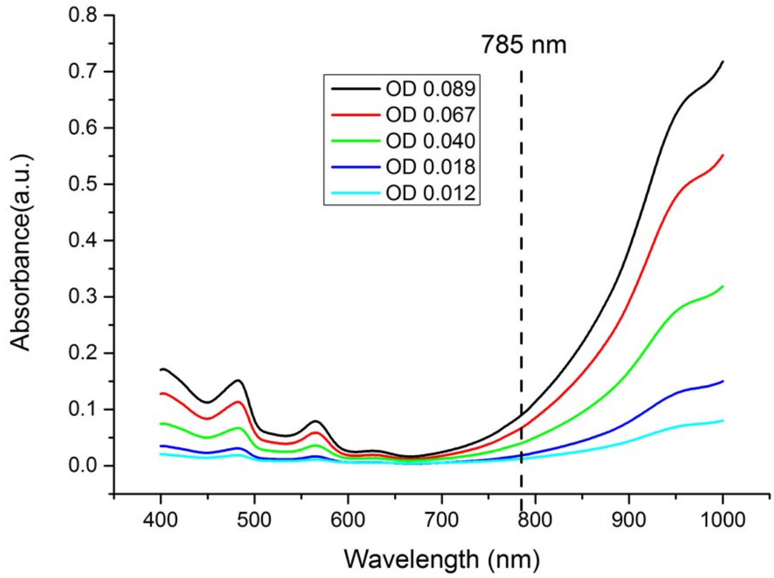
785 nm
OD 0.089
OD 0.067
OD 0.040
OD 0.018
OD 0.012
Absorbance(a.u.)
0.0
0.1
0.2
0.3
0.4
0.5
0.6
0.7
0.8
400
500
600
700
800
900
1000
Wavelength (nm)

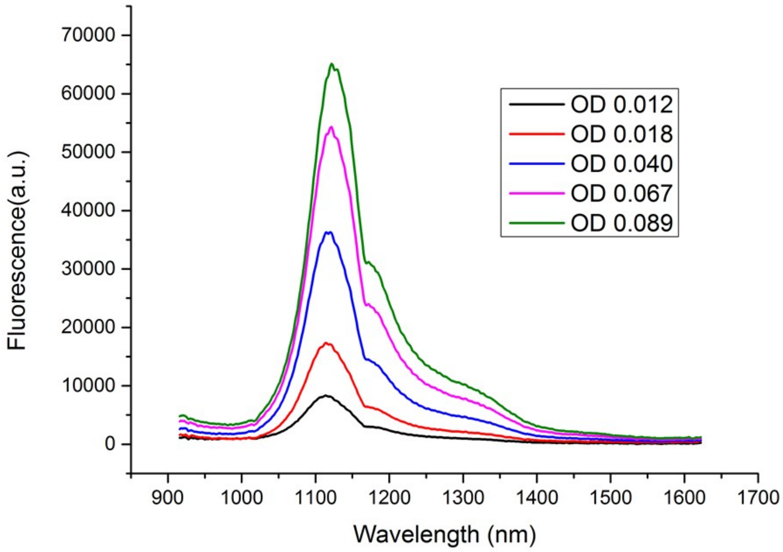
OD 0.012
OD 0.018
OD 0.040
OD 0.067
OD 0.089
Fluorescence(a.u.)
0
10000
20000
30000
40000
50000
60000
70000
900
1000
1100
1200
1300
1400
1500
1600
1700
Wavelength (nm)

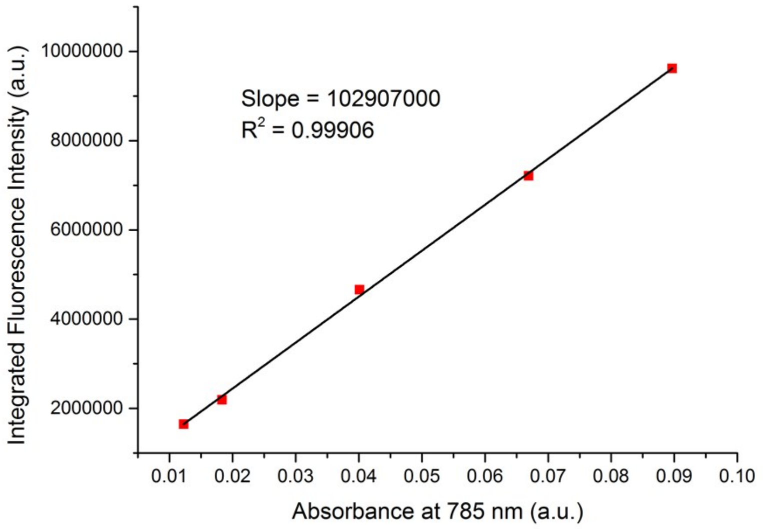


The fluorescence quantum yield measurement was carried out using IR-26 whose quantum yield has been reported as 0.5% in 1,2-dichloroethane (DCE) as reference. In a typical procedure, a series of solutions of IR-26 in DCE with absorbance values at 785 nm to be ~ 0.10, ~ 0.08, ~ 0.06, ~0.04 and ~0.02 were prepared respectively. Optical absorbance spectra of the five solutions were measured using PerkinElmer Lambda 25 UV-Vis spectrophotometer. NIR emission spectra of the five solutions with linearly spaced concentrations were measured using Applied NanoFluorescence spectrometer. The 785 nm laser was used as the excitation source and a 900 nm long-pass filter was used as the emission filter to acquire the emission spectrum in the range of 900 to 1600 nm. Same solution preparation, absorbance and fluorescence spectra measurements were performed for **H1**.

## References


1. A. L. Antaris, et al. *Nat. Mater.*, **2016**, *15*. 235–242.
2. Y. Sun, et al. *Chem. Sci.*, **2016**, *7*.6203-6207.
3. E. Perzon, et al. *Polymer*, **2006**, *47*. 4261-4268.
4. E. Perzon, et al.*Adv. Mater.*,**2007**, *19*. 3308-3311.
5. V. C. Marhevka, et al. *J. Med. Chem.*, **1985**, *28*. 18–24.

**NMR Spectra**

## [1]H for Compound 2

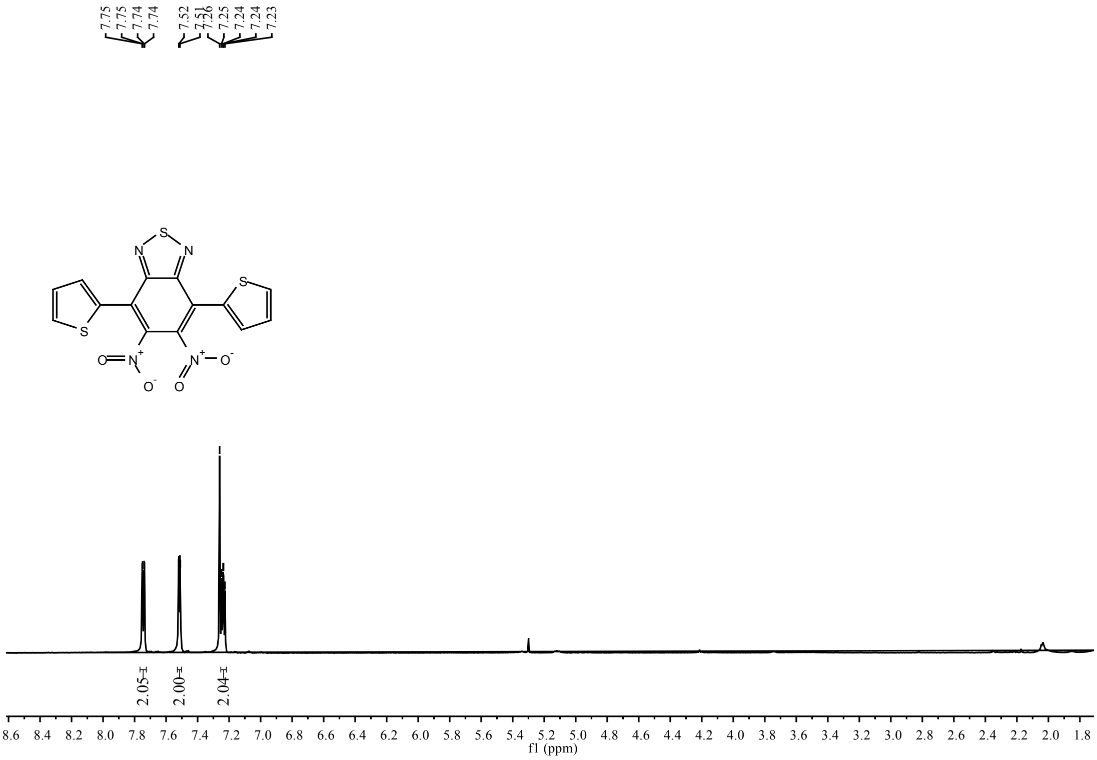


## [1]H for Compound 3

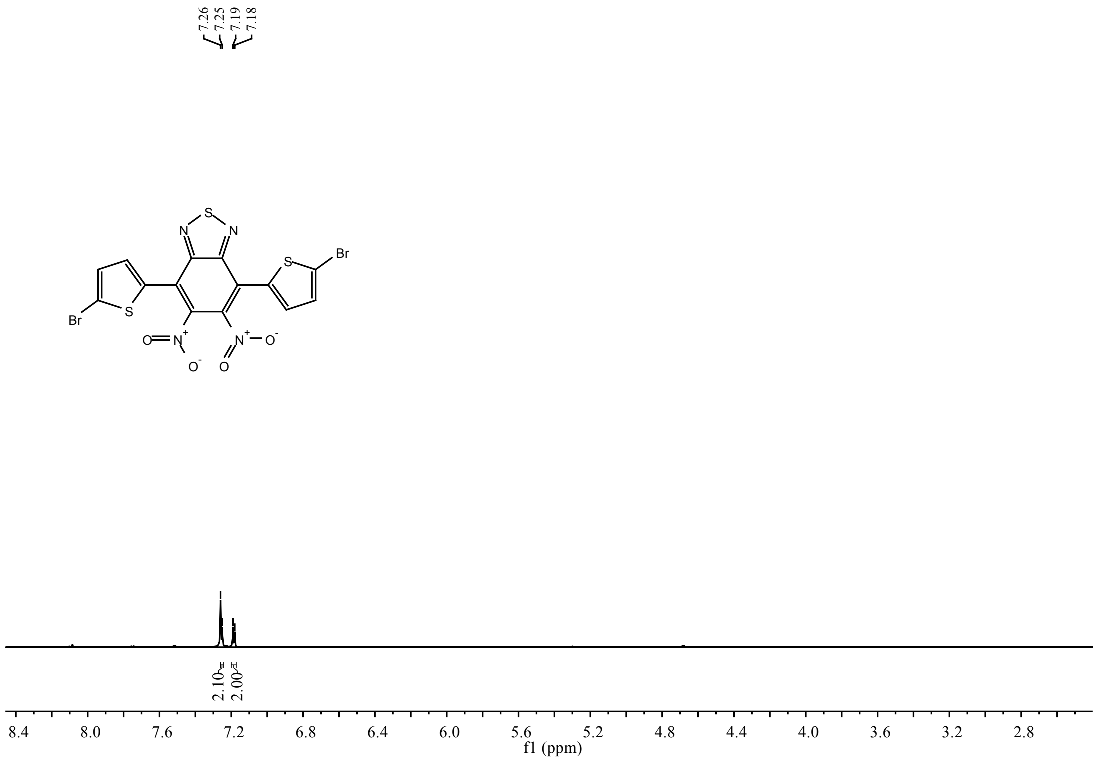

## $^1$H for Compound 4-1

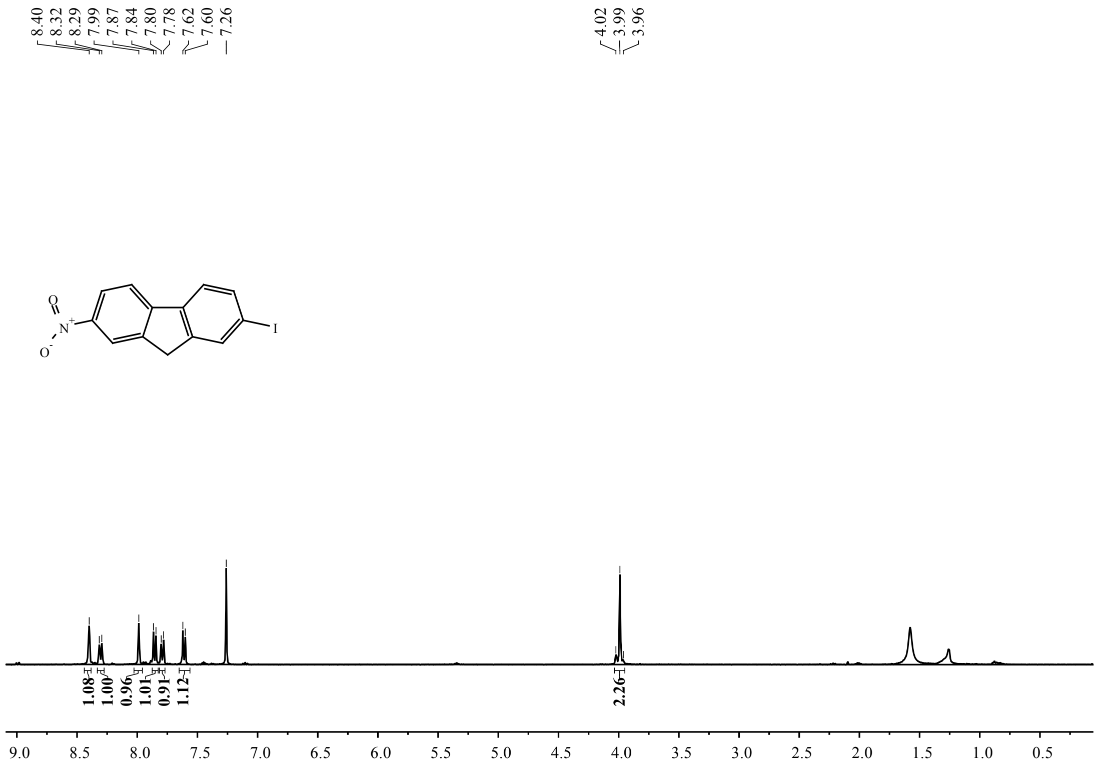


## $^1$H and $^{13}$C NMR for Compound 4-2

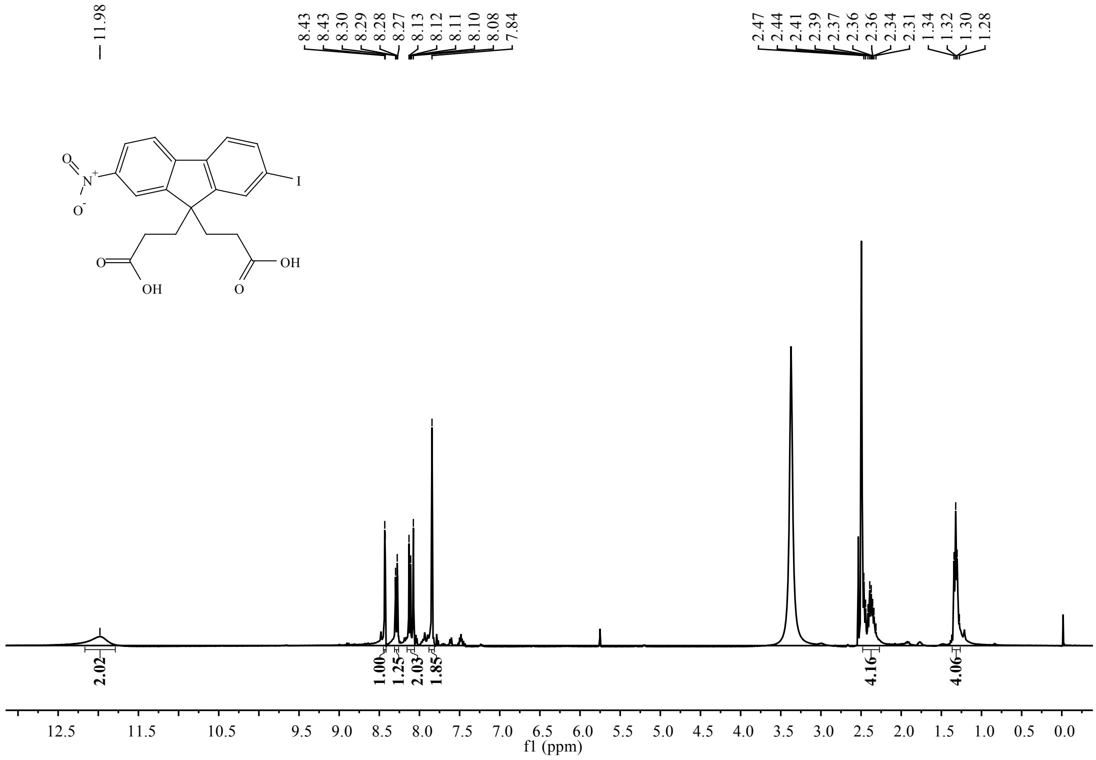

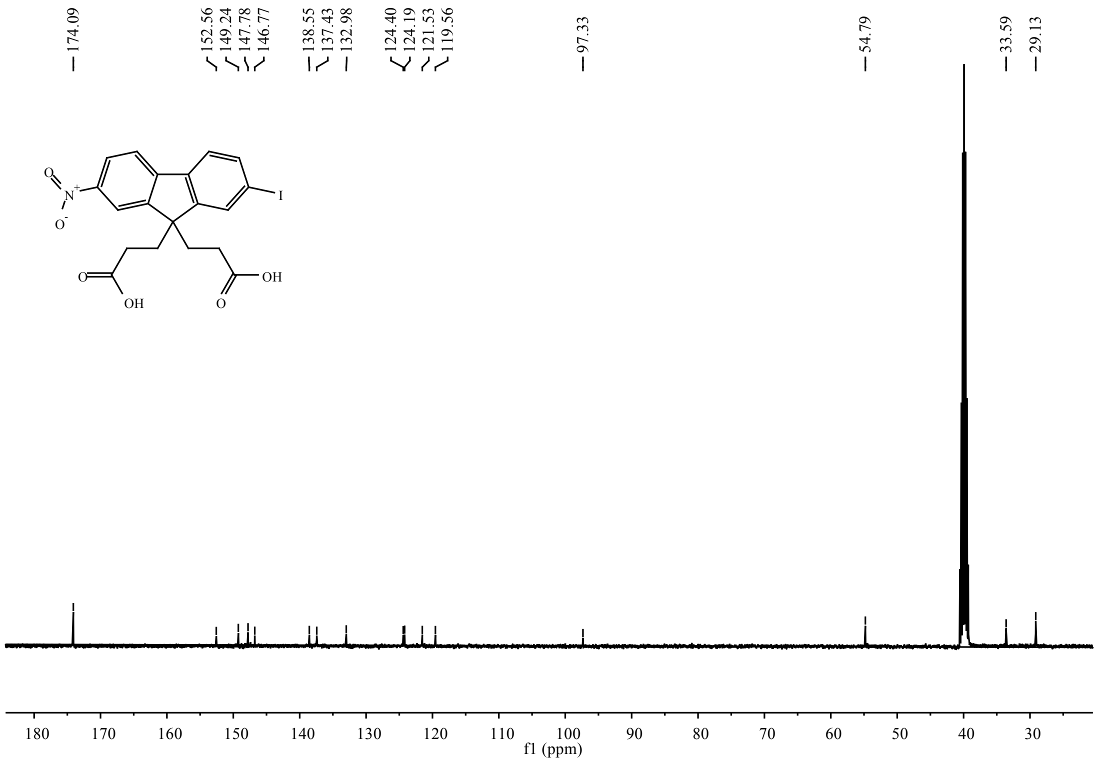


# $^{1}$H and $^{13}$C NMR for Compound 4-3

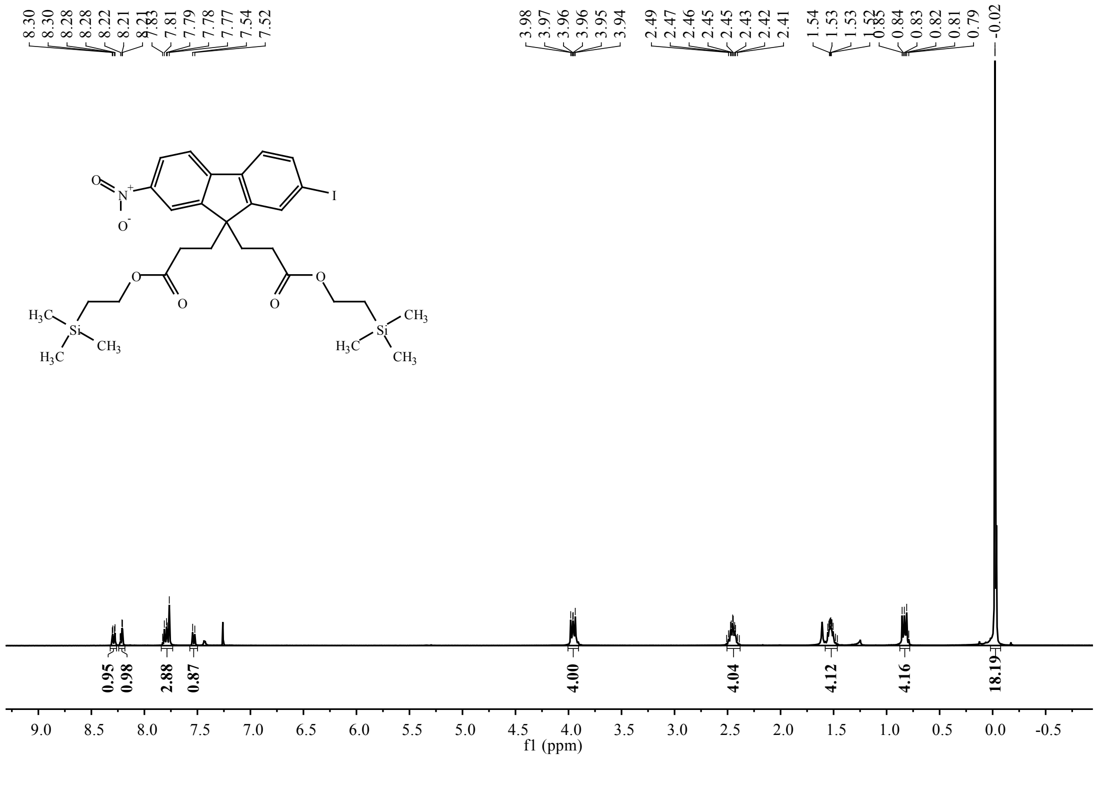

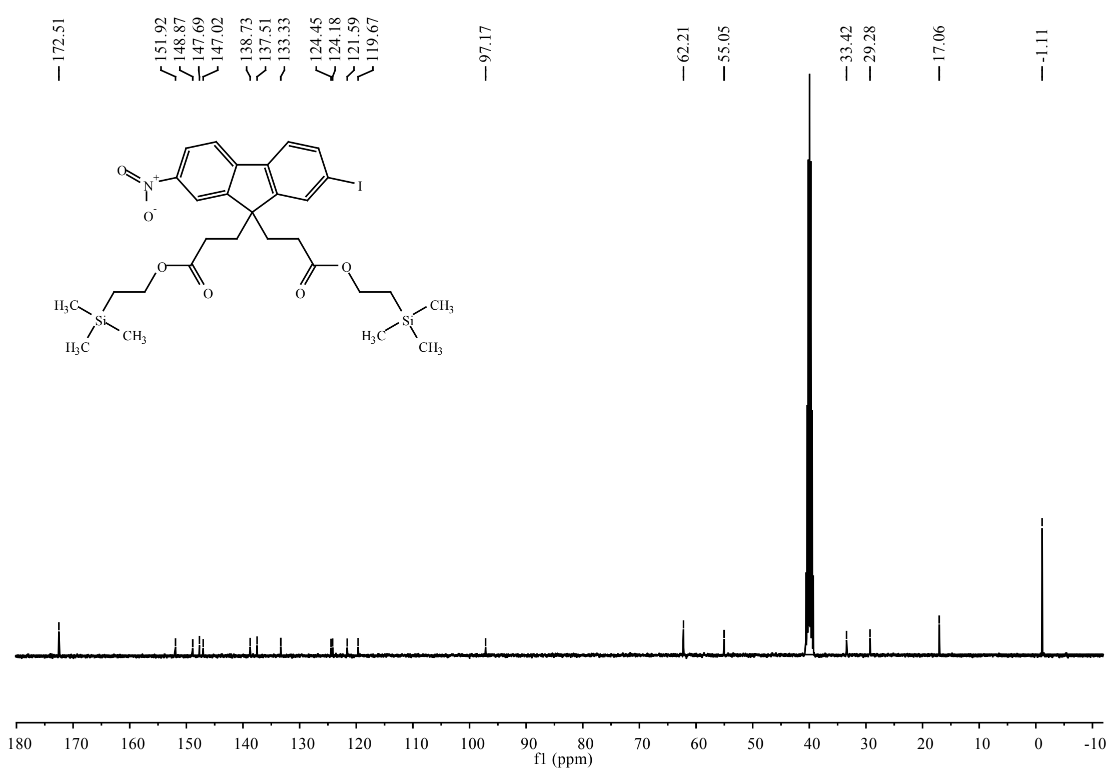

# $^{1}$H and $^{13}$C NMR for Compound 5

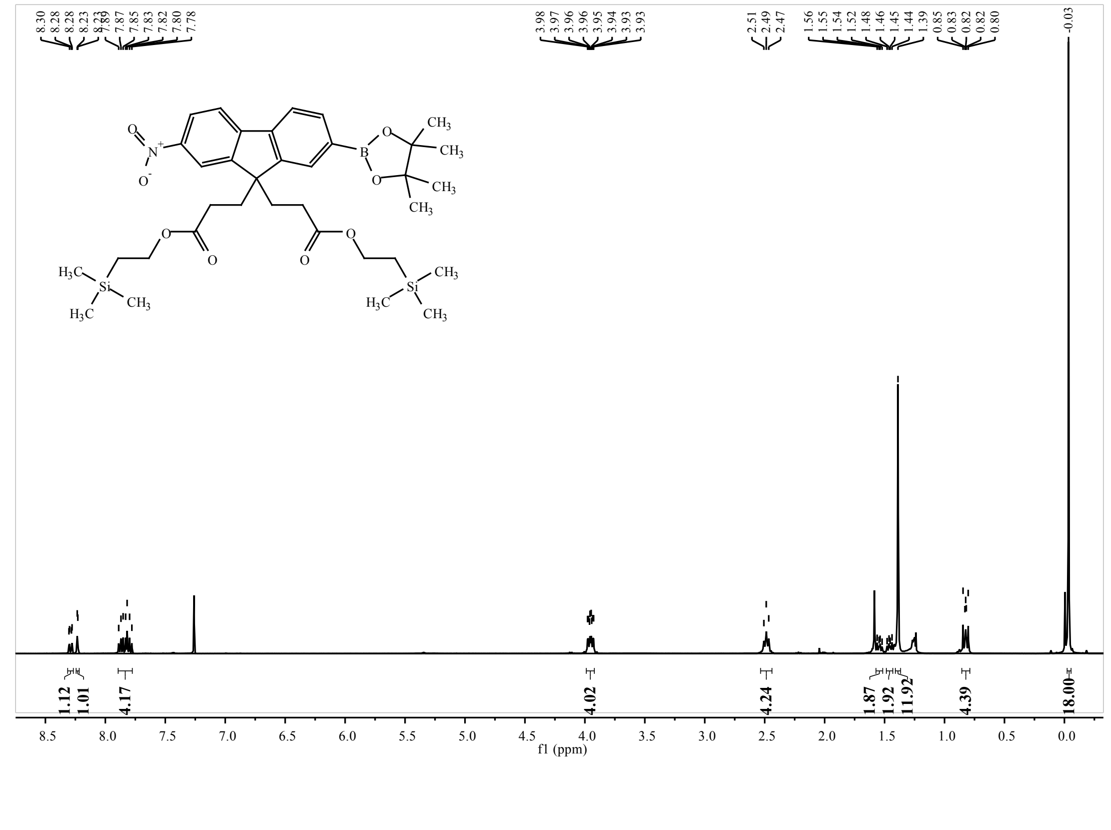

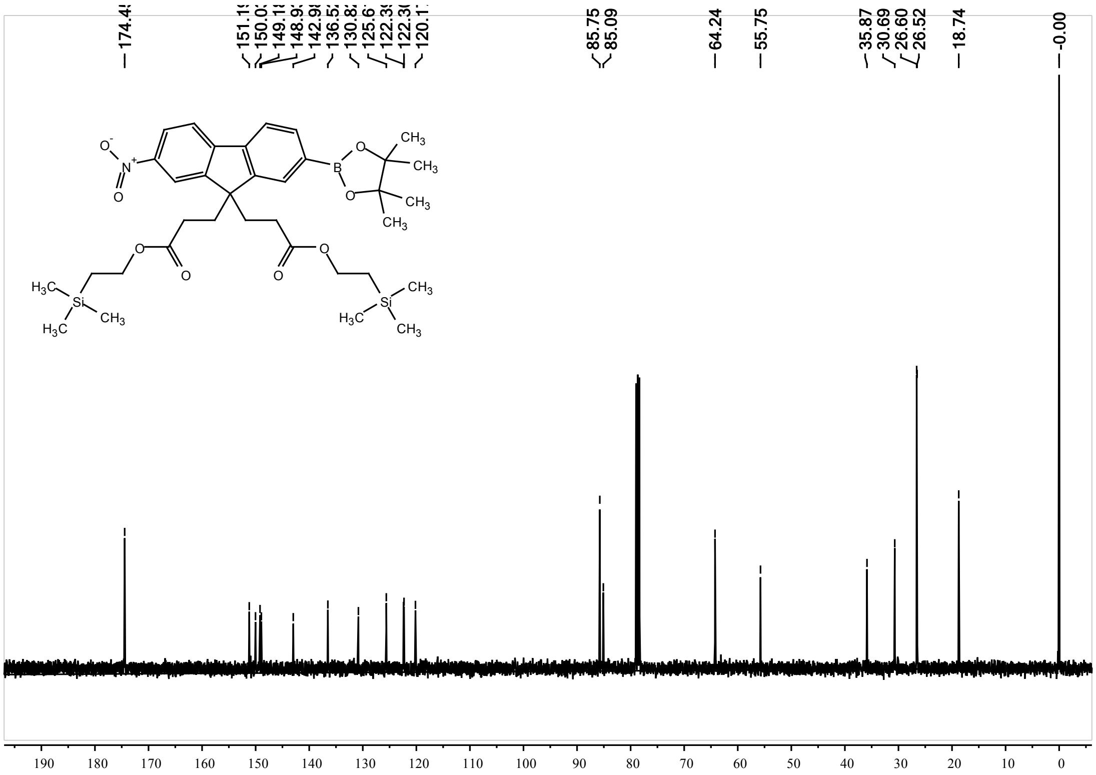


## $^{1}$H and $^{13}$C NMR for Compound 6

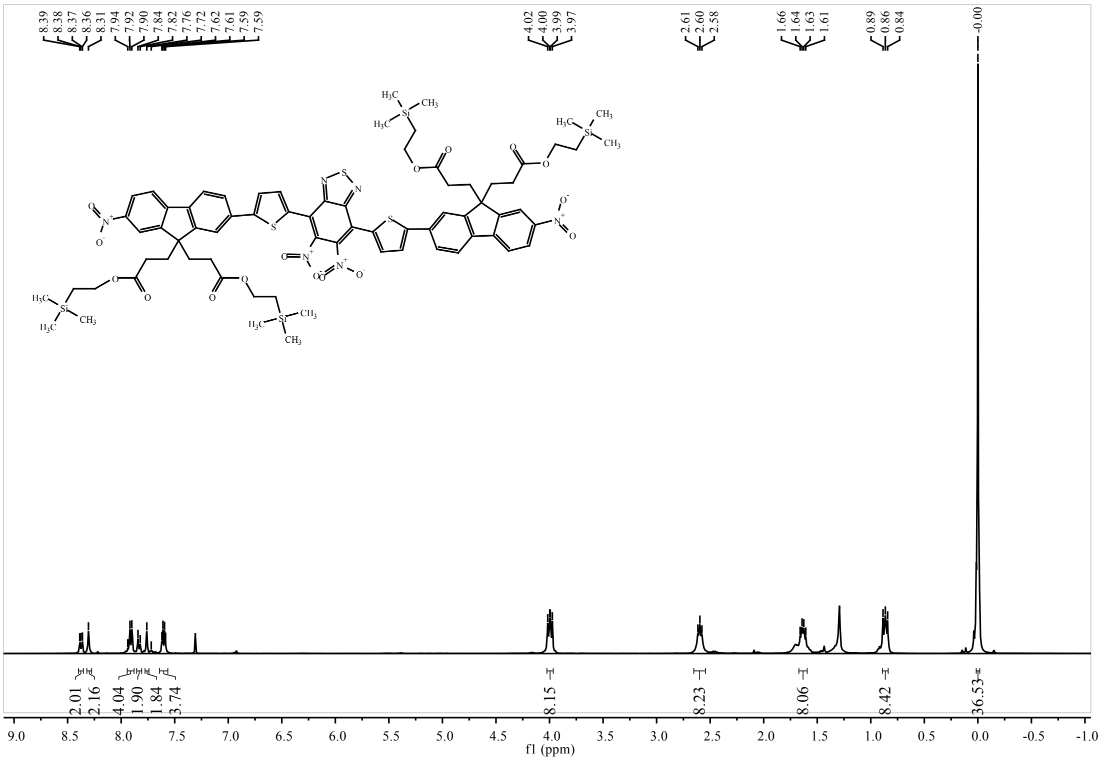

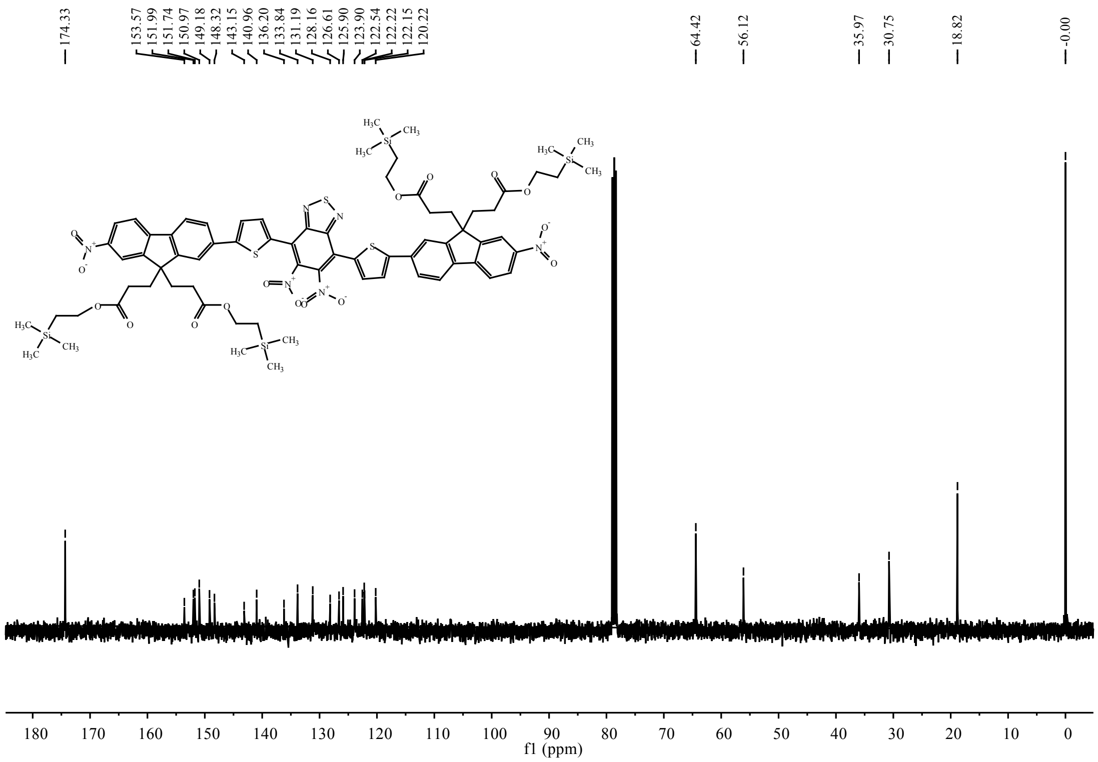


## $^1H$ and $^{13}C$ NMR for H1

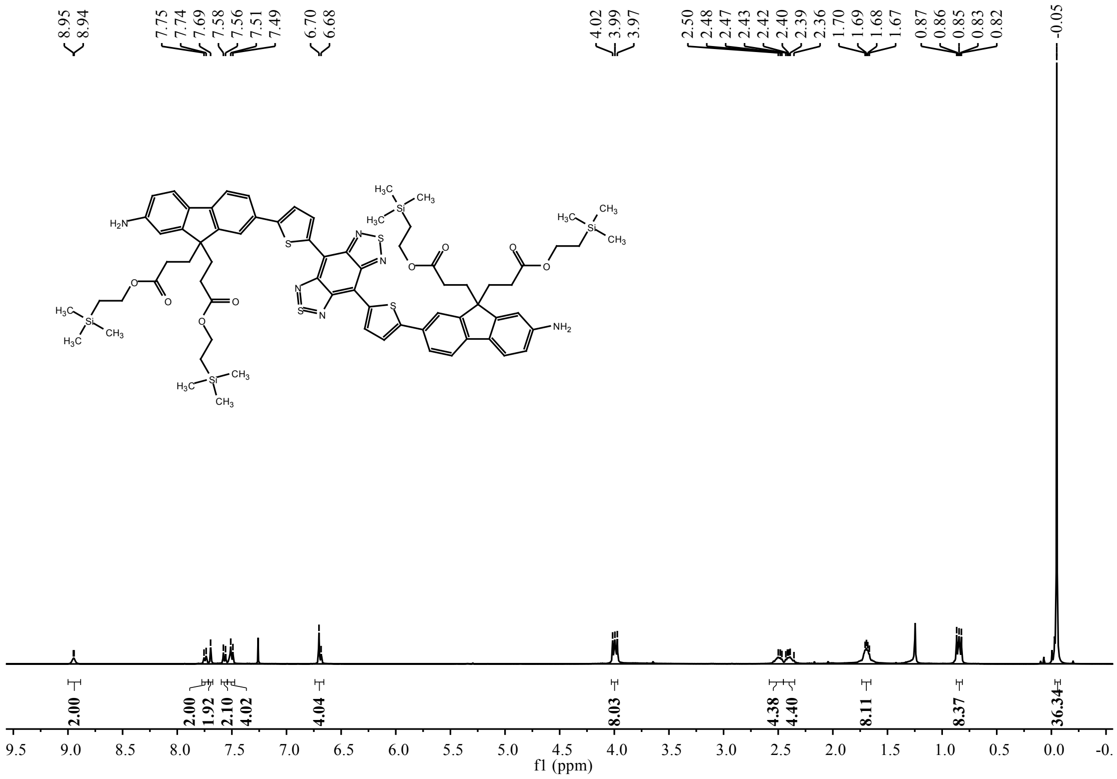

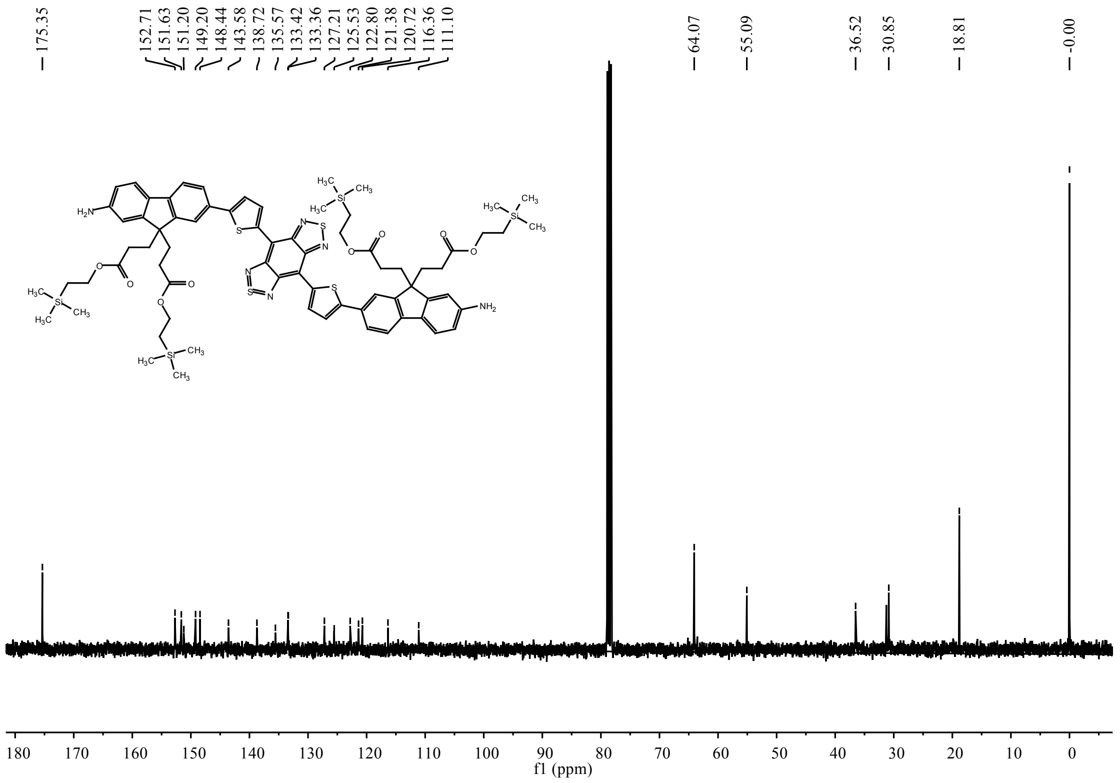
175.35
152.71
151.63
151.20
149.20
148.44
143.58
138.72
135.57
133.42
133.36
127.21
125.53
122.80
121.38
120.72
116.36
111.10
64.07
55.09
36.52
30.85
18.81
-0.00
180 170 160 150 140 130 120 110 100 90 80 70 60 50 40 30 20 10 0
f1 (ppm)

**MS Spectra**

**MALDI-TOF-MS for Compound H1**

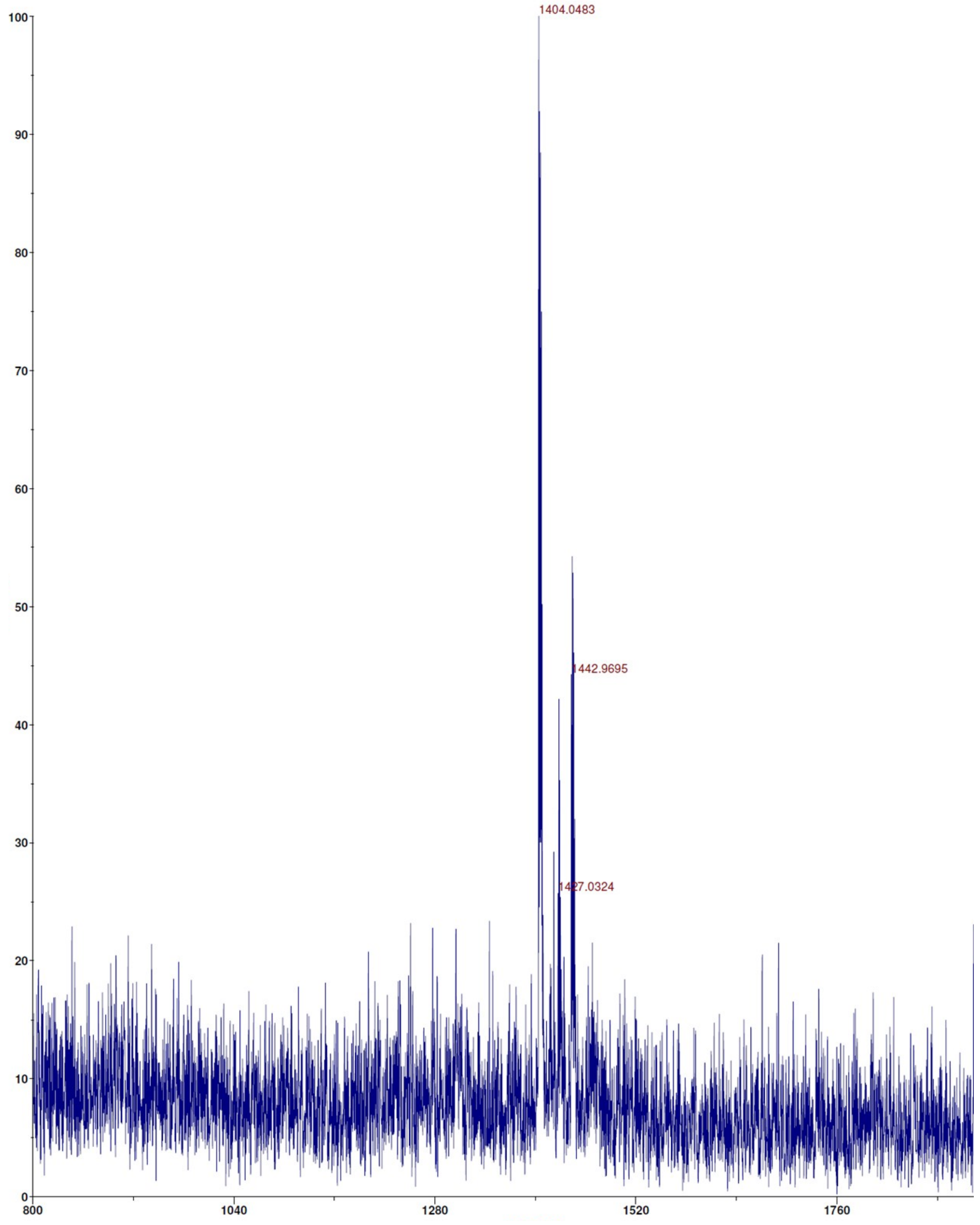

**MALDI-TOF-MS for Compound 7**

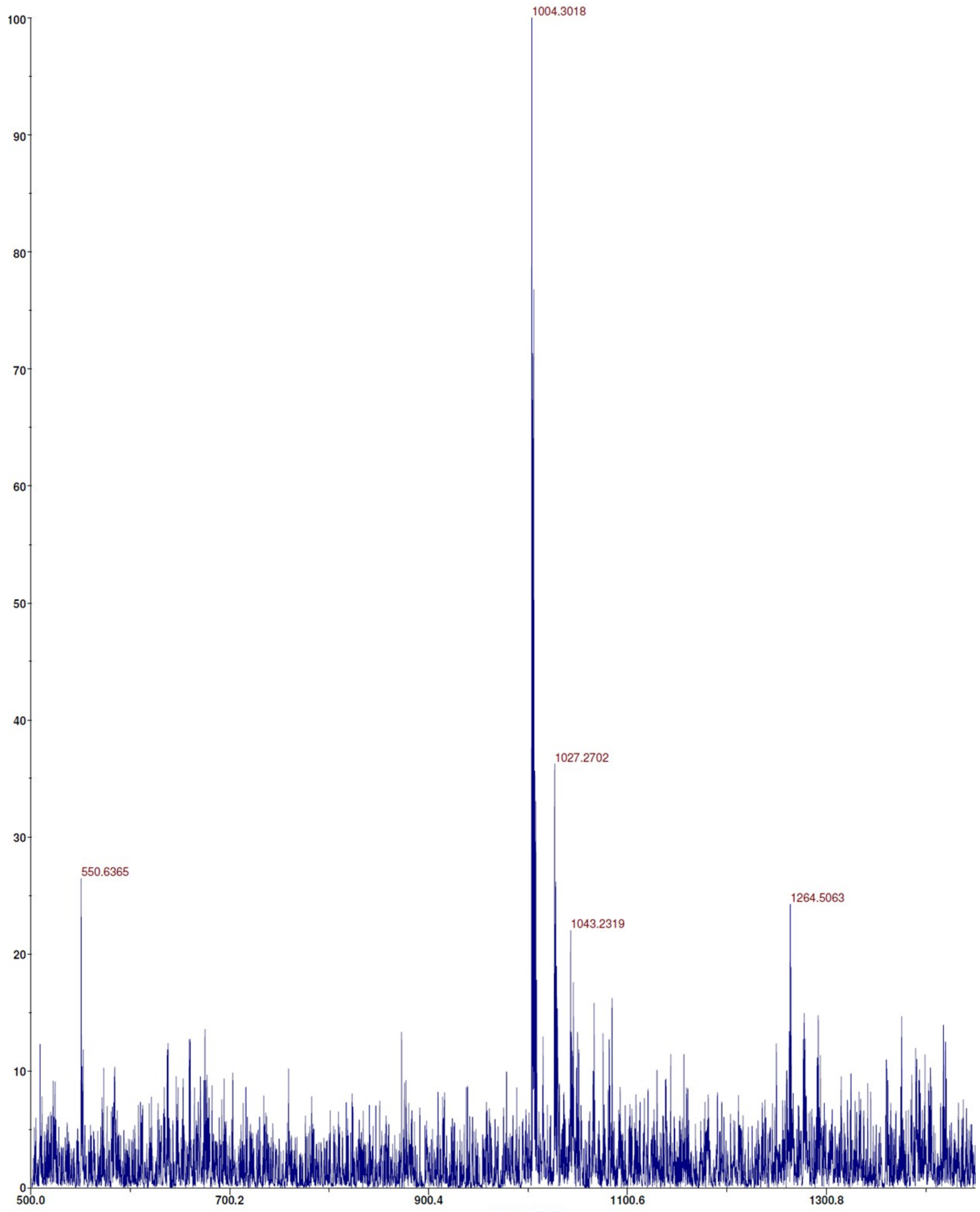

## MALDI-TOF-MS for SXH

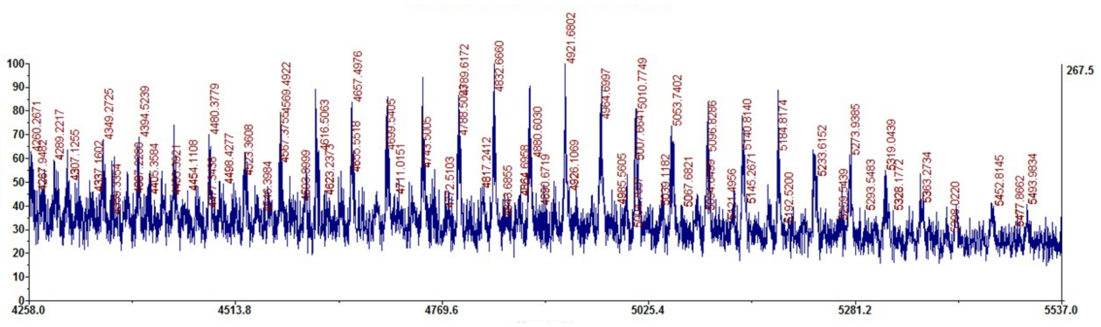

**MALDI-TOF-MS for SDH**

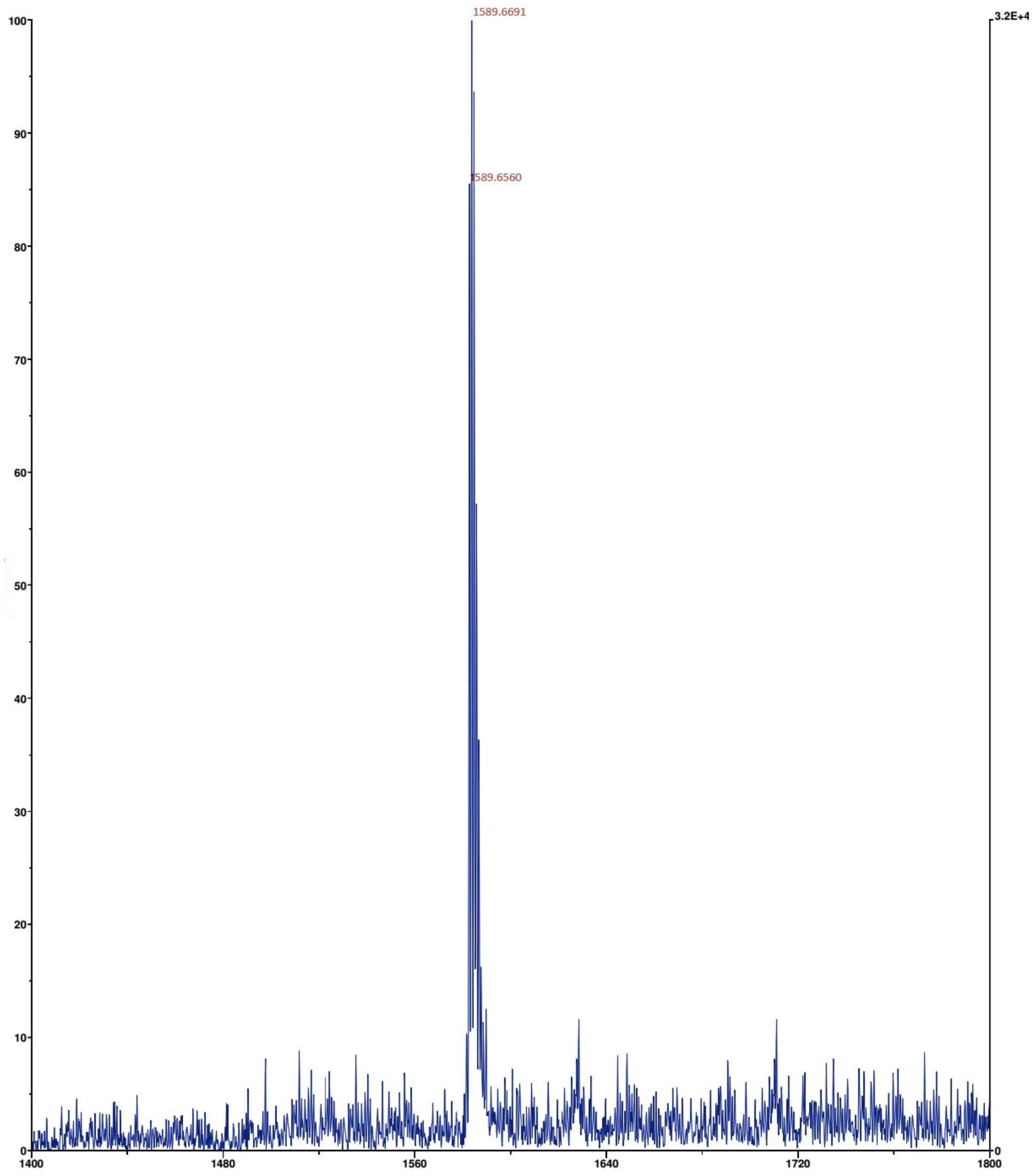

Supplement: Supplementary file 1 [file SC-008-C7SC00251C-s001.pdf]
